# Occupancy time in sets of states for demographic models

**DOI:** 10.1016/j.tpb.2017.12.007

**Published:** 2018-03

**Authors:** Gregory Roth, Hal Caswell

**Affiliations:** Institute for Biodiversity and Ecosystem Dynamics, University of Amsterdam, Netherlands

**Keywords:** Matrix population models, Absorbing Markov chains, Multistage models, Demography, Occupancy time, Longevity, Southern fulmar

## Abstract

As an individual moves through its life cycle, it passes through a series of states (age classes, size classes, reproductive states, spatial locations, health statuses, etc.) before its eventual death. The occupancy time in a state is the time spent in that state over the individual’s life. Depending on the life cycle description, the occupancy times describe different demographic variables, for example, lifetime breeding success, lifetime habitat utilisation, or healthy longevity.

Models based on absorbing Markov chains provide a powerful framework for the analysis of occupancy times. Current theory, however, can completely analyse only the occupancy of single states, although the occupancy time in a set of states is often desired. For example, a range of sizes in a size-classified model, an age class in an age×stage model, and a group of locations in a spatial stage model are all sets of states.

We present a new mathematical approach to absorbing Markov chains that extends the analysis of life histories by providing a comprehensive theory for the occupancy of arbitrary sets of states, and for other demographic variables related to these sets (e.g., reaching time, return time). We apply this approach to a matrix population model of the Southern Fulmar (*Fulmarus glacialoides*). The analysis of this model provides interesting insight into the lifetime number of breeding attempts of this species.

Our new approach to absorbing Markov chains, and its implementation in matrix oriented software, makes the analysis of occupancy times more accessible to population ecologists, and directly applicable to any matrix population models.

## 1 Introduction

The life of an individual is a sequence of events. Birth and death are events common to every individual, but the sequence between birth and death – unique to each individual – consists of a potentially endless list of random events (e.g., surviving, developing, mating, reproducing, growing, dispersing, moving among social or occupational classes, or changing health status). Each event corresponds to a change in the state of the individual, resulting in a stochastic pathway that ends eventually in death. A central role in the analysis of these pathways is played by the concepts of *occupancy time* (the time spent in, or the number of visits to, a state over the individual’s lifetime). Occupancy is a property of the stochastic pathway of an individual, and occupancy times define the time spent in each of the possible states during the lifetime. In particular, the longevity of an individual is measured by the sum of all these occupancy times. The interpretation of occupancy times depends on the identity of the transient states and the nature of the absorption. Thus, when the states are health status, occupancy time represents years of life while healthy, not healthy, disabled, etc. When the states are spatial locations, occupancy time represents time spent in different places. When the states are marital status, occupancy times measure the part of the lifetime spent single, married, divorced, remarried, etc. When the states are employment status, or breeding activities, or any other interesting categorisation of individuals, the interpretation follows the same lines. As for absorption, it may be death, in which case occupancy time is a “lifetime” measure in the literal sense. But absorption can be defined as the first entrance to some state or set of states (e.g., occurrence of first breeding, or graduation, or metamorphosis, or hospitalisation, etc.).

Because the pathways are stochastic, occupancy time is a random variable. It is often described by its mean (e.g., life expectancy, expected lifetime reproduction). However, some individuals will live longer and some shorter, than the mean; some will mature later and some earlier than the mean. To characterise this variation, the probability distribution of occupancy time, or at least its moments, must be considered.

Models based on absorbing Markov chains provide a powerful framework for the analysis of occupancy times. An absorbing Markov chain describes the fate of an individual – under the assumption that the future of the individual, given its present, is independent of its past – evolving in a set of states and being eventually absorbed by the death state. The states may refer to developmental states, physiological measures, behaviourtypes, locations, and so on. The set of transition rates between these states – described by a *transition matrix* – defined an absorbing Markov chain. As a population projection matrix describes the fate of a population, the transition matrix describes the fate of individuals in a population, and often is one component of a population projection matrix. The mathematical theory of absorbing Markov chain provides formulae for basic descriptive quantities of the absorbing Markov chain, based on its fundamental matrix (see e.g. Iosifescu (1980) and Kemeny and Snell (1961) for a mathematical perspective, and Caswell (2001) for a demographical perspective). Applied to demographic models, this theory provides simple and direct formulae for the probability distribution, the mean, variance, and all moments of longevity, the distribution of age or stage at death, the survivorship and mortality functions, causes of death, and a variety of measures of life disparity (e.g., [11], [9], [1], [2], [3], [26], [12], [28]). Powerful sensitivity analyses are available for all these quantities [2], [3], [5], [6].

Current theory, however, can completely analyseonly the occupancy time of single states and the occupancy time of the whole state space. Our goal is to extend the analysis of life histories by providing a comprehensive theory for the occupancy of arbitrary sets of states. One type of set is a collection of states deemed biologically relevant for some purpose; we call these *super-states*. For example, a model based on reproductive behaviour might include states describing many details of the success, failure, timing, and number of offspring produced by breeding, but one might want to investigate the super-states created by aggregating these into “successful breeding” and “non-successful breeding” sets. A spatial model might describe habitats along an altitudinal gradient, and one might want to aggregate in order to compare the occupancy of low altitude and high altitude sites. A medical demography study might distinguish a variety of health conditions and treatments, but one might want to compare the occupancy of all states requiring hospitalisation and those not requiring hospitalisation. The utility of super-states will increase as more matrix models are created from the growth and survival kernels of integral projection models (e.g., Ellner et al., 2016). These matrices typically contain hundreds of size classes, no one of which is of particular interest, but sets of which (e.g., all trees large enough to reach the forest canopy) are of great interest.

A second type of sets of states arises in the context of multistate (or megamatrix) or hyperstate models (e.g., [19], [16], [18], [27], [20]) in which individuals are classified by two or more criteria (age and stage, stage and location, etc.). One may want to analyse the occupancy of sets of states defined by integrating over one of these criteria; we call these *marginal sets*. For example, in a stage×size-classified model, the marginal set associated with the *juvenile* stage is the set containing the juvenile stage, integrated over all possible sizes.

The extension of occupancy time calculations to sets of states may seem trivial because the occupancy time in a set is the sum of the occupancy times in each state belonging to this set. Therefore, the mean occupancy time in a set is the sum of the means of the occupancy times in each state. However, this observation does not hold for the variance, for any higher moments, or for the probability distribution, because occupancy times in single states are not *independent* from each other. There are few analyses of the occupancy time in set of states, but they only focus on specific aspects of it. For example, Steiner and Tuljapurkar (2012) provide formulae for the mean and variance of the reproductive output using the joint generating function of the single state occupancy times. The reproductive output of an individual is closely related to the occupancy time in the set of reproductive states (both are equal when fertility rates are ones in each reproductive state). Caswell (2011a) provides similar formulae using the theory of Markov chain with reward. The same theory is used by Caswell and Kluge (2015) to calculate the moments of lifetime accumulation of economic variables, which are also closely related to occupancy times. However, these studies do not provide the probability distribution of occupancy time in a set of states. In the mathematical literature, Sericola (2000) provides an iterative formula for the probability distribution of the partial (i.e. up to a fixed time) occupancy time in a set of states, but does not provide a closed formula for the total occupancy time. Here, we present a comprehensive approach to calculate the any moment and the probability distribution of the occupancy time in arbitrary sets of states. Our approach relies on the construction of a *sub Markov chain*, which describes the original Markov chain viewed through a filter that allows one to see only the states in the set of interest. As a consequence, all the statistics of the occupancy time in the set of interest may be calculated with the existing theory of absorbing Markov chain (Iosifescu, 1980), applied to the sub Markov chain.

The construction and the analysis of the sub chain extends the classical theory of absorbing Markov chain by providing not only several measures related to the occupancy of sets of states but also forms a basis for further calculations of measures related to sets of states, including

• The set occupancy time. Depending on the life cycle description, set occupancy times describe different demographic variables (e.g., lifetime breeding attempts in a model of reproductive behaviour, or lifetime habitat utilisation in a spatial model). We provide for the probability distribution, mean, and variance of the occupancy times.
• The correlation between the occupancy times in two different sets. This is an indicator of how the two sets are connected in the life cycle. As a particular case, we provide, for the first time, a formula for the correlation between the occupancy time in a state and the longevity of an individual. Depending on the life cycle description, this formula gives the correlation between different demographic variables and longevity (e.g., lifetime breeding attempts and longevity, lifetime reproduction and longevity, time to maturation and longevity).
• Properties of winners and losers. Relative to a particular target set, a *winner* is an individual that enters the set at least once in its life, and a *loser* is an individual that never enters the set. In a model classifying individuals by their developmental state, the winners might represent those individuals that successfully mature, and the losers those that do not. We provide the probability of becoming a winner, the distribution, mean, and variance of the time required for a winner to reach the set, and the longevity of a loser. After its first success, a winner may leave the set and never return, or it may return at some future time. We obtain the probability that a winner returns, and for those that do return, the probability distribution, mean, and variance of the return time.

Table 1 lists the demographic results to be presented and the equations in which they are derived. All the results are obtained directly from a single matrix, describing the transition probabilities among transient states. This matrix is obtainable from most population projection matrices (Caswell, 2001). Despite the large number of matrices and sometimes complicated expressions that appear in our derivations, our results are easily computed in matrix-oriented software. In the Supplementary Material, we provide the Matlab code for calculating all of the demographic results listed in Table 1.

### Notation

Matrices are denoted by upper-case bold symbols, vectors by lower-case bold symbols. Vectors are column vectors by default. When a matrix has a subscript (e.g., $U_{K}$), we note its entries with a superscript, $u_{ij}^{K}$. The vector $1_{\xi }$ is the ξ×1 vector of ones, and the vector $0_{\xi }$ is the ξ×1 vector of zeros. The matrix $I_{\xi }$ is the identity matrix of size ξ×ξ. The transpose of A is $A^{\text{T}}$. The diagonal matrix with the vector z on its diagonal is diag(z). The Hadamard product (component by component) of A and B is A∘B. Any random variable x is defined on a probability space with probability P, its expectation is denoted by E[x] and its variance is denoted by Var(x). The kth moment, $E\left[y^{k}\right]$, of a random vector y is denoted by $y^{k}$.

**Table 1:** Results of the set analysis in a Markov chain demographic model.

Output	Expression	Equation
Occupancy time in B		

Mean and variance	$E\left[\tau_{B}\right],\mathrm{Var}\left(\tau_{B}\right)$	(20), (24), (25), (26)
Moments	$\tau_{B}^{k}$	(23)–(25)
Distribution		(28)–(29)

Reaching the set B		

Probability to reach	$p_{a}$	(39)
Time to reach:	$t_{B}$	(41)
Mean and variance	$E\left[t_{B}\right],\mathrm{Var}\left(t_{B}\right)$	(43)–(44)
Moments	$t_{B}^{k}$	(42)
Distribution		(45)

Returning to the set B		

Probability to return	$p_{r}$	(47)
Time to return:	μ	(48)
Mean and variance	E[μ],Var(μ)	(53)–(54)
Moments	$\mu^{K}$	(97), in the appendix
Distribution		(51)–(52)

Correlation between the occupancy times in two sets		

Correlation	$\mathrm{Corr}\left(\tau_{B_{1}},\tau_{B_{2}}\right)$	(86)–(36)

Correlation between the occupancy time in B and the longevity		

Correlation	$\mathrm{Corr}\left(\tau_{B},\eta \right)$	(38)

## 2 Absorbing Markov chains as demographic models

An absorbing Markov chain describes the fate of a particle – under the assumption that the future of the particle, given its present, is independent of its past – evolving in a set of states and being eventually absorbed by an absorbing state. In a demographic model, the absorbing Markov chain describes the fate of an individual evolving through a set of states and being absorbed by the state representing its death. The absorbing Markov chain is determined by two key elements: the living states of the individuals, and the transition probabilities between those states, which depend on the entire set of vital rates.

Formally, we consider the finite set of living states T={1,…,ω}, and the inevitable state d, representing death. The living states and the death state are called respectively the *transient states* and *absorbing state* of the Markov chain. The state space of the Markov chain is their union,

(1) S=T∪{d}.

An absorbing Markov chain is uniquely defined by the one-step transition probabilities and an initial probability distribution. The transition probabilities are described by the matrix P of size (ω+1)×(ω+1). For each i,j∈S, the i−j entry of P is the probability that an individual in state j moves to state i. Therefore, the columns of P sum to one (i.e. P is a stochastic matrix). Note that in the mathematical literature, the transition matrix is given by the transpose of P; however, our notation is widely used in the demographical literature. The matrix P can be decomposed in four blocks: (2)
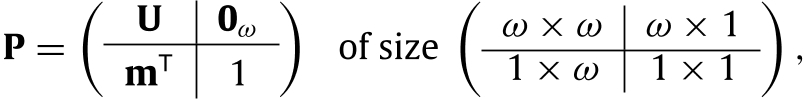
where the matrix U of size ω×ω is the *transient transition matrix*, which describes the transition probabilities between the transient states, and the column vector m of size ω contains the *probabilities of death* from each transient state.

Since the column sums of P are equal to one, the column sums of matrix U are less than one (strictly less than one if the death probabilities are assumed to be non zero). Moreover, since there is a single absorbing state, the vector m is in fact a function of the matrix U,

(3) $$m^{\text{T}}=1_{\omega }^{\text{T}}-1_{\omega }^{\text{T}}U.$$

As a consequence, the matrix P is function of the transient transition matrix U, which is the only variable of the model.

### An absorbing Markov chain from a population projection matrix

A population projection matrix can often be decomposed in two parts: one is the matrix U that describes the transitions of living individuals and the other is a matrix F that describes the rates of production of new individuals (see e.g. Caswell (2001)). Therefore, the population projection matrices contain an absorbing Markov chain, which is defined by the transient transition matrix U.

### Target states

The focus of this paper is the occupancy time in a set of living states, which we call the *target set*. To fix the idea, we define a target set B composed of β transient states, and we re-number the states so that the target states are the last β elements of T, i.e. 

B={α+1,α+2,…,ω}

$$B^{c}=\left\{1,2,\ldots ,\alpha \right\},$$

where α=ω−β. According to this renumbering of the states, we re-arrange the entries of the matrix P. This results in a new matrix U and a new vector m. To avoid overloaded notation, we shall retain the symbols U and m. It will be clear from the context which arrangement is being used.

Iosifescu (1980) derives formulae for the moments of the occupancy time of the Markov chain in a *single* target state (see also Caswell (2009)). The key element for those calculations is the *fundamental matrix*

(4) $$N={\left(I_{\omega }-U\right)}^{-1}.$$

The i-jth entry of the matrix N is the mean occupancy time in the state i for an individual starting in state j. Any higher moment of the occupancy time is derived from the fundamental matrix. In particular, the variances are given by the matrix,

(5) $$V=\left(2I_{\omega }\circ N-I_{\omega }\right)N-N\circ N.$$

The mean occupancy time in the target set is the sum of the mean occupancy times in each target state. Hence, the mean is directly deduced from the fundamental matrix by summing the corresponding entries. However, this technique breaks down when calculating the variance of the occupancy time. Indeed, the variance of a sum of random variables is not the sum of the variance of each variable (unless the variables are independent). This complication motivates the need of the new technique presented in the next Section. This technique provides not only a straightforward formula for the variance, but also an entire set of new formulae for diverse measures of life history traits, as listed in Table 1.

### Example

**Fig. 1:**
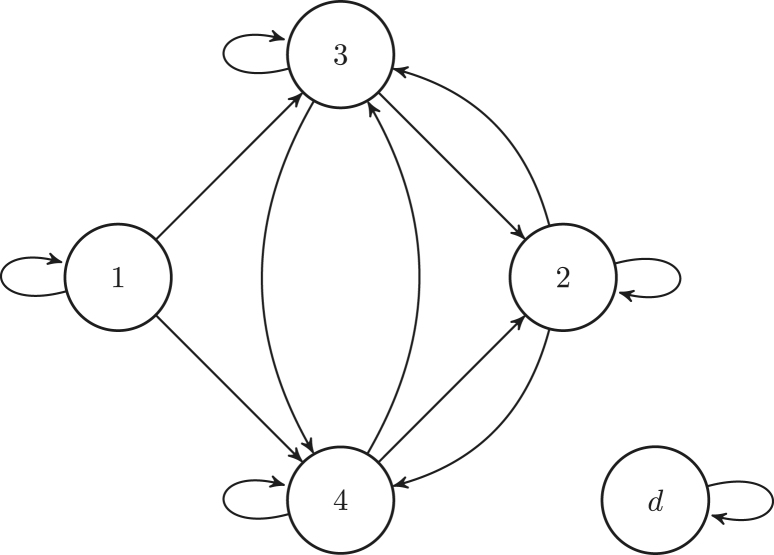
Life cycle graph of Southern Fulmar. State 1 is the pre-breeder state. State 2 is the non-breeder state. State 3 is the successful breeder state. State 4 is the failed breeder state. State d is the death state. To lighten the graph we omit the transitions to the death state.

The model and the results are illustrated throughout the paper with a model for the Southern Fulmar (*Fulmarus glacialoides*) derives by Jenouvrier et al. (2015). The life cycle graph shown in Fig. 1 is broken into four stages, which are defined at the end of the breeding season: (1) pre-breeders, who have yet to breed for the first time; this include fledged chicks from the previous season; (2) non-breeders, who have bred at least once before, but not in the current season; (3) successful breeders, who have successfully raised a chick during the current season, and (4) failed breeders, who have not successfully raised a chick during the current season because they failed to either hatch an egg or raise a chick. Hence, the set of transient states is T={1,2,3,4} and the individual state space is S={1,2,3,4}∪{d}. We define two target sets: $B_{a}=\left\{\text{2, 3, 4}\right\}$ (β=3 and α=1) and $B_{b}=\left\{\text{3, 4}\right\}$ (β=2 and α=2). The former represents the super-state *adult*, that is an individual in $B_{a}$ is characterised by having bred at least once. The latter represents the super-state *breeding*; an individual in $B_{b}$ is currently attempting to breed regardless of its success.

### Outcome of a Markov chain model

An outcome of a demographic Markov chain model is function of the initial state of an individual, and its pathway through the life cycle. For example, the number of breeding attempt of a newborn Southern Fulmar is the result of its entire pathway — from birth to death. Here, the initial state is pre-breeder because any newborn is in this state. The number of breeding attempt of an adult is a result of its pathway, but only from its first passage in the super-state adult to death. Here, the initial state is successful breeder (or failed breeder) because an individual matures when it reaches one of these two states. For this reason, any result listed in Table 1 is a vector and each entry corresponds to a specific initial condition. For example, the occupancy time in B is given by the vector

(6) $$\tau_{B}=\left(\tau_{1},\ldots ,\tau_{\omega }\right)$$

where $\tau_{i}$ is the occupancy time in the set B for an individual that is initially in state i.

## 3 Constructing the induced Markov chains

To derive our results, we construct three new Markov chains associated with the set of target states B. These chains are created from the *original* Markov chain P and the target set B.

• The *killed Markov chain*, with transition matrix $P_{K}$. This Markov chain is a copy of the original Markov chain that is stopped as soon as it enters in the target set. If the individual never enters the target set, then the pathway of the killed Markov chain is equivalent to the pathway of the initial Markov chain. See Section 3.1 for a formal definition.
• The *conditional Markov chain*, with transition matrix $P_{C}$. This Markov chain concerns only individuals that successfully reach the target set. It describes their pathway from their initial state to their first entrance to the target set. In other words, it describes these pathways of the killed Markov chain that do reach a target state. See Section 3.2 for a formal definition.
• The *sub-Markov chain*, with transition matrix $P_{S}$. This Markov chain is a copy of the original Markov chain viewed through a filter that allows one to see only the target set. The pathways of the sub-Markov chain corresponds to the pathways of the original Markov chain observed through this filter. See Section 3.3 for a formal definition.

**Fig. 2:**
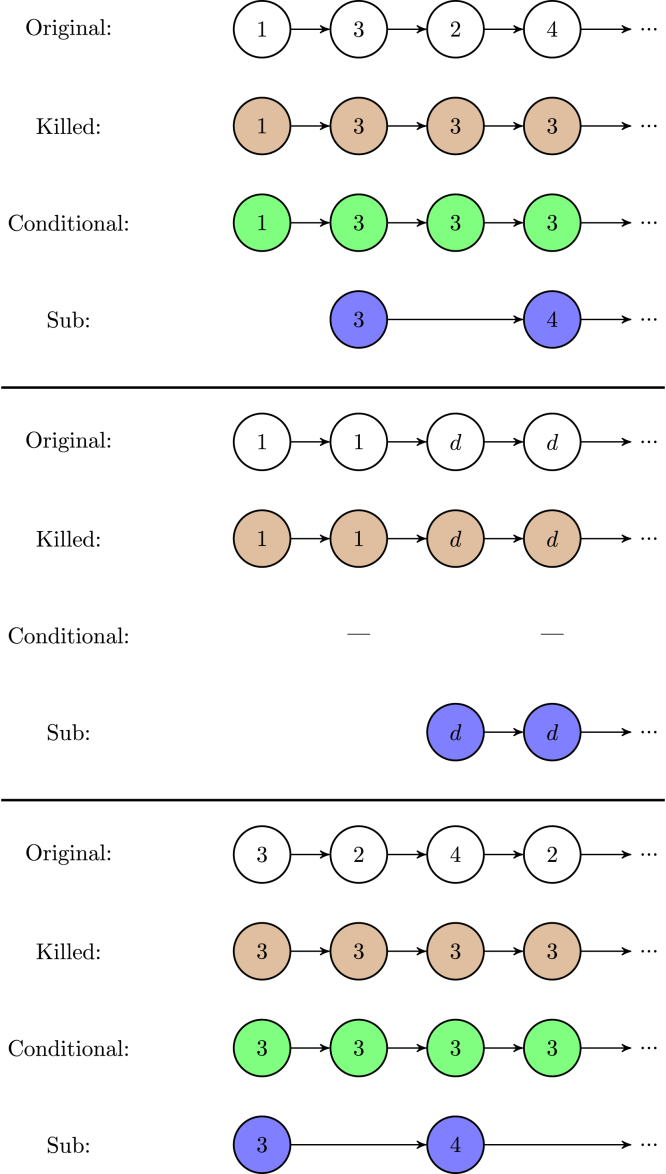
The first row of each block is a realisation of the original Markov chain describing the Southern Fulmar life cycle. Under them are the corresponding realisations of the induced Markov chains associated with the target states {3,4}: the killed Markov chain in brown, the conditional Markov chain in green, and the sub-Markov chain in blue. The realisations of the sub chain may have longer jumps because it describes the pathways of the original chain only in the target set. In the second block, the conditional chain has no realisation because the original chain does not enter any target state. (For interpretation of the references to colour in this figure legend, the reader is referred to the web version of this article.)

To each pathway of the original Markov chain there corresponds a pathway in each induced Markov chain. Fig. 2 shows three pathways through the life cycle of the Southern Fulmar, and, below each of them, the corresponding pathways of the induced Markov chains, associated with the target set $B_{b}$, consisting of the breeding states.

We now explain in detail how to construct the matrices $P_{K}$, $P_{C}$, and $P_{S}$ from the matrix P; we illustrate this construction with the Southern Fulmar example. After the rearrangement of the matrix P according to the numbering of the states (see Section 2), the transition matrix U can be split in four block matrices, which contain the transition probabilities from $B^{c}$ to $B^{c}$, from $B^{c}$ to B, from B to $B^{c}$, and from B to B, respectively. These block matrices are denoted by $U_{K}$, L, K, and Q, and appear within U as Eq. (7) given in (Box I).

These four matrices are essential to the construction of the killed, the conditional, and the sub Markov chains. All the other matrices used for this construction and for the derivation of the results are tabulated for easy reference in Table 2.

**Table 2:** Matrices used the construction and in the analysis of the Markov chain demographic model. ω and β denote the number of living states in the life cycle and the number of states in the set B, respectively, and α=ω−β.

Notation	Expression	Size	Description
Matrices describing demographic model			

P		(ω+1)×(ω+1)	Transition probabilities
U		ω×ω	Living states transition probabilities
m		ω×1	Mortality probabilities
N	${\left(I_{\omega }-U\right)}^{-1}$	ω×ω	Fundamental matrix

Decomposition of U			

$U_{K}$	Eq. (7)	α×α	$B^{c}$-to-$B^{c}$ transitions
K	Eq. (7)	α×β	$B^{c}$-to-B transitions
L	Eq. (7)	α×β	B-to-$B^{c}$ transitions
Q	Eq. (7)	β×β	B-to-B transitions

Matrices describing the killed MC			

$P_{K}$		(ω+1)×(ω+1)	Transition probabilities
$U_{K}$		α×α	Transient state transition probabilities
$M_{K}$	Eq. (9)	(β+1)×α	Absorbing transition probabilities
$N_{K}$	${\left(I_{\alpha }-U_{K}\right)}^{-1}$	α×α	Fundamental matrix

Matrices describing the conditional MC			

$P_{C}$		(α+1)×(α+1)	Transition probabilities
$U_{C}$	$D_{a}U_{K}D_{a}^{-1}$	α×α	Transient state transition probabilities
$m_{C}$	${\left(1_{\alpha }^{\text{T}}KD_{a}^{-1}\right)}^{\text{T}}$	α×1	Absorbing transition probabilities
$N_{C}$	${\left(I_{\alpha }-N_{C}\right)}^{-1}$	α×α	Fundamental matrix

Matrices describing the sub MC			

$P_{S}$		(α+1)×(α+1)	Transition probabilities
$U_{S}$	AL+Q	α×α	Transient state transition probabilities
$m_{S}$	$1_{\beta }^{\text{T}}-1_{\beta }^{\text{T}}U_{S}$	1×α	Mortality probabilities
$N_{S}$	${\left(I_{\beta }-U_{S}\right)}^{-1}$	β×β	Fundamental matrix

Other			

A	$KN_{K}$	β×α	Reaching a state in B probabilities
$\overset{\sim }{A}$	$\left(A|I_{\beta }\right)$	β×ω	
$p_{a}$	$A^{\text{T}}1_{\beta }$	α×1	Reaching B probabilities
$D_{a}$	$\text{diag}\;\left(p_{a}\right)$	α×α	
$p_{r}$	$U_{S}^{\text{T}}1_{\beta }$	β×1	
$D_{r}$	$\text{diag}\;\left(p_{r}\right)$	β×β	

Transition probabilities given individual returns in B			

$W_{\mathrm{in}}$	$QD_{r}^{-1}$	β×β	B to B transitions
$W_{\mathrm{out}}$	$D_{a}LD_{r}^{-1}$	α×β	B to $B^{c}$ transitions

Box I (7)
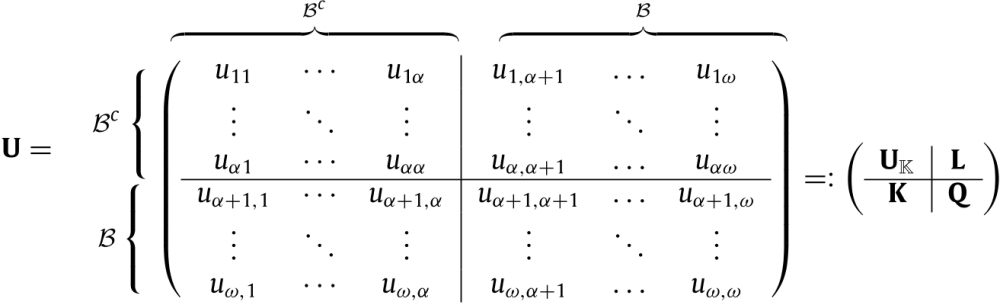

### 3.1 The killed Markov chain $P_{K}$

The transition matrix $P_{K}$ describes a copy of the original Markov chain that is stopped as soon as it enters B. In this chain, the states in B and the death state d are now absorbing, and the new transient set is $B^{c}$. The state space of the killed Markov chain is S, and its transition probability matrix is

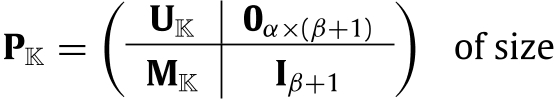

(8)
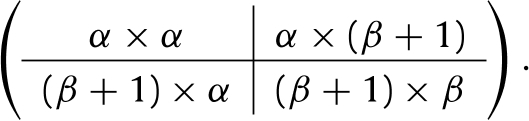

The entry $u_{ij}^{K}$ is the transition probability from non-target state j to non-target state i. The entry $m_{ij}^{K}$ is the transition probability from state j to target state α+i, for i=1,…,β, and to the death state, for i=β+1. The matrix $U_{K}$ is extracted from the original transition matrix U, as in Eq. (7). The matrix $M_{K}$ is composed of two blocks: (9)
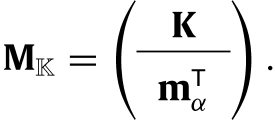
The matrix K describes the transitions from the non target states to the target states; it is directly extracted from the original transition matrix U, as in Eq. (7). The vector $m_{\alpha }$ describes the transitions from the non target states to the death states; it is extracted from the original vector m describing the probabilities of death (Eq. (2)),

(10) $$m_{\alpha }=\left(m_{1},\ldots ,m_{\alpha }\right).$$

#### Example

**Fig. 3:**
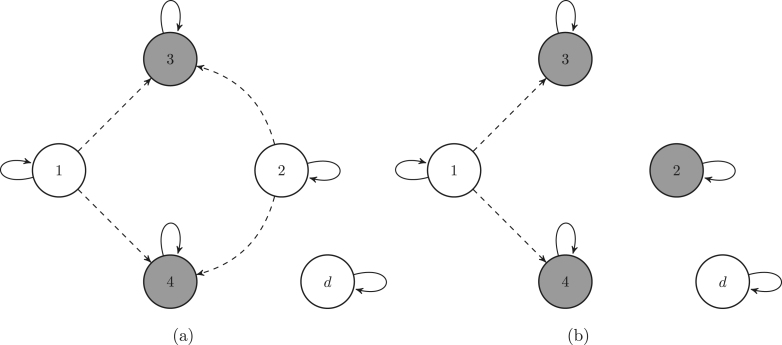
Graphs of the killed Markov chain associated with the sets {3,4} (a), and {2,3,4} (b), respectively. The grey circles are the target states, which are absorbing states for the killed Markov chain. The dashed arrows are the transitions into the absorbing states. To lighten the graph we omit the transitions to the death state.

Fig. 3 shows the graphs of the killed Markov chains associated with the target states $B_{b}=\left\{3,4\right\}$ and $B_{a}=\left\{2,3,4\right\}$, respectively.

#### 3.1.1 Absorption probabilities

In order to define the conditional Markov chain, we need to calculate, for each target state i and non-target state j, the probability that an individual starting in j passes through i before eventual death. For j=1,…,α, and i=1,…,β, let $a_{ij}$ denote the probability that the killed chain initially in state j (non target state) is absorbed into the target state α+i. Following Theorem 3.3 in Iosifescu (1980), those probabilities are described by the matrix

(11) $$A=KN_{K}\;\;\text{ of size }\beta \times \alpha ,$$

where $N_{K}={\left(I_{\alpha }-U_{K}\right)}^{-1}$ is the fundamental matrix associated with the killed Markov chain. Note that the matrix A is a sub-matrix of the matrix $A^{*}=M_{K}N_{K}$, which describes the entire distribution of fates for any starting non-target state, i.e. the probabilities that the killed Markov chain is stopped in any of the target states or in the death state. Therefore the columns of $A^{*}$ sum to one.

The probabilities of absorption in the set B – regardless of the specific state in which the chain is absorbed – are given by the vector $p_{a}$ which satisfies the equation

(12) $$p_{a}^{\text{T}}=1_{\beta }^{\text{T}}A.$$

The ith entry of $p_{a}$ is the probability that the killed Markov chain, initially in state i, is absorbed in a target state.

By definition of the killed Markov chain, $a_{ij}$ is the probability that the first target state, visited by an individual initially in state j, is α+i. Similarly, $p_{j}^{a}$ is the probability that an individual initially in state j reaches a target state. In the Southern Fulmar example with target set $B_{b}=\left\{3,4\right\}$, the entry $a_{11}$ is the probability that the first breeding attempt of a newborn is successful, and $p_{1}^{a}$ is the probability that a newborn attempts breeding.

### 3.2 The conditional Markov chain $P_{C}$

The transition matrix $P_{C}$ describes the transition probabilities of the killed Markov chain, conditional on absorption in the target set. Therefore, the absorbing states are B and the new transient set is $B^{c}$. The state space of the conditional Markov chain is T, and its transition probability matrix is (13)
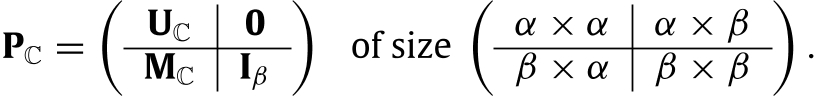
Note that the death state does not belong to the state space of the conditional Markov chain because all its trajectories are absorbed by the target set before death is reached. The entry $u_{ij}^{C}$ is the conditional probability of the transition form state j to state i given that the individual will eventually enter in the set B. The entry $m_{ij}^{C}$ is the conditional transition probability from the state j to the target state α+i, given that the individual will eventually enter in the set B. The columns of $P_{C}$ sum to 1. The block matrices $U_{C}$ and $M_{C}$ are given by

(14) $$U_{C}=D_{a}U_{K}D_{a}^{-1}\;\text{ and }\;M_{C}=KD_{a}^{-1},$$

where

(15) $$D_{a}=\text{diag}\;\left(p_{a}\right)$$

is a diagonal matrix with, on the diagonal, the probabilities of absorption in the target states, $p_{a}$ (defined in Eq. (11)).

In Appendix A.2, we provide a formal proof that the matrix $P_{C}$, defined by Eqs. (13), (14), describes the Markov chain whose trajectories are precisely those of the killed Markov chain that do not encounter death before entering the target set. This proof is deeply inspired by the proof, written by Iosifescu (1980) (Section 3.2.9), in the special case where the target set is a single state.

#### Example

**Fig. 4:**
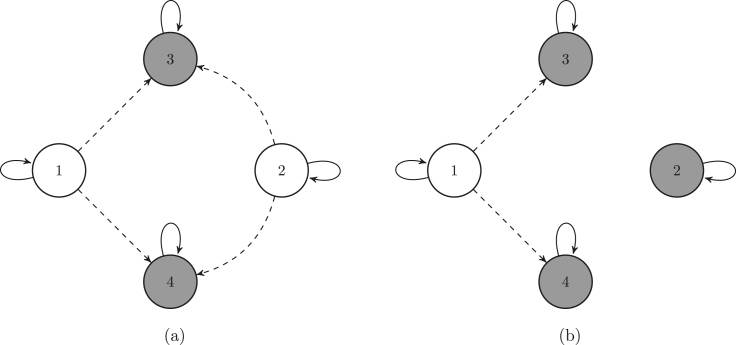
Graphs of the conditional chain associated with the sets {3,4} (a), and {2,3,4} (b), respectively. The grey circles are the target states. The dashed arrows are the transitions into the absorbing states.

Fig. 4 shows the graphs of the conditional Markov chains associated with the target states $B_{a}=\left\{3,4\right\}$ and $B_{a}=\left\{2,3,4\right\}$, respectively.

### 3.3 The sub-Markov chain $P_{S}$

The transition matrix $P_{S}$ contains, for each pair of target states i and j, the probabilities that an individual currently in state i returns to the target set in state j or dies before returning to the target set. Note that the return may take more than one time step. The transient state space is B, and d is the only absorbing state. The state space of the sub-Markov chain is B∪{d}. The transition probability matrix is (16)
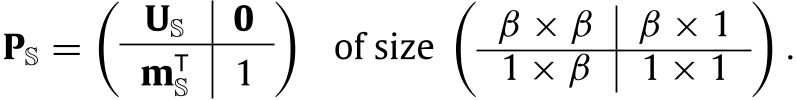
The entry $u_{ij}^{S}$ is the probability that an individual starting in state α+j reaches the state α+i without passing through any other state in B. The entry $m_{i}^{S}$ is the probability that an individual starting in state α+i dies before it reaches a state in B. Since the columns of the matrix $P_{S}$ sum to one,

(17) $$m_{S}^{\text{T}}=1_{\beta }^{\text{T}}-1_{\beta }^{\text{T}}U_{S},$$

and the only variable is the matrix $U_{S}$. In Appendix A.1, we derive the matrix $U_{S}$ from the matrix P,

(18) $$U_{S}=AL+Q\;\;\text{ of size }\beta \times \beta .$$

The matrix Q (defined in Eq. (7)) contains the probabilities that an individual starting in a target state makes a one-step transition to a target state. The product AL describes the probabilities that the individual first leaves, and then re-enters, the target set. The matrix L is defined in Eq. (7), and the matrix A is defined in Eq. (11).

Note that one time-step for the sub-Markov chain corresponds to a random number of time-steps for the initial Markov chain. However, the number of passages in a specific state in B starting in B is equivalent for both chains (see Section 4 for more details).

#### Example

**Fig. 5:**
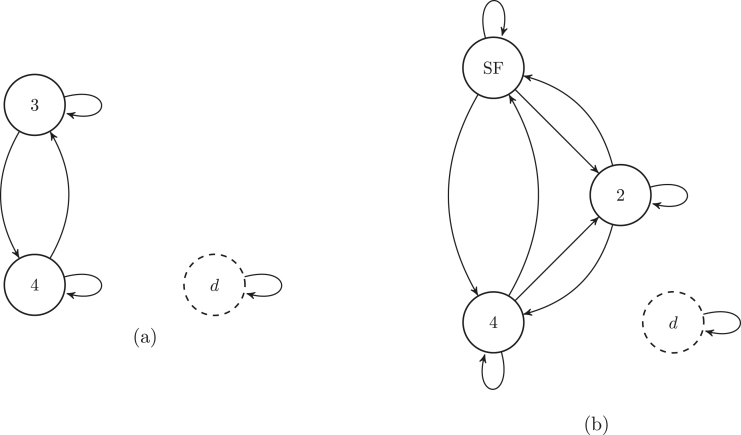
Graphs of the sub-Markov chain associated with the sets {3,4} (a), and {2,3,4} (b), respectively. The dashed arrows are the transitions into the absorbing states. The dashed circles are the target states. To lighten the graph we omit the transitions to the death state.

Fig. 5 shows the graphs of the conditional Markov chains associated with the target states $B_{a}=\left\{3,4\right\}$ and $B_{b}=\left\{2,3,4\right\}$, respectively.

## 4 Occupancy time in the target states

The occupancy time in the target states is the time spent in the target states over the individual’s life. Because the pathways taken by individuals through their life is stochastic, the occupancy time is a random variable; each of its realisation is associated with the realised pathway of an individual. In this section, we calculate the distribution of the occupancy time in B and we provide formulae for its mean and variance, and for any of its higher moments.

Let $\tau_{i}$ denote the occupancy time in the set B for an individual initially in state i. The occupancy times are grouped into two vectors, $\tau_{\mathrm{in}}$ and $\tau_{\mathrm{out}}$, depending on the initial state being in or out of the set B, and both of the vectors are concatenated in the vector $\tau_{B}$,

(19) $$\tau_{B}={\left(\underset{≕\tau_{\mathrm{out}}}{\underset{︸}{\tau_{1},\ldots ,\tau_{\alpha }}},\underset{≕\tau_{\mathrm{in}}}{\underset{︸}{\tau_{\alpha +1},\ldots ,\tau_{\omega }}}\right)}^{\text{T}}.$$

Let $\tau_{B}^{k}$, $\tau_{\mathrm{out}}^{k}$, and $\tau_{\mathrm{in}}^{k}$ denote the kth moment of the random vectors $\tau_{B}$, $\tau_{\mathrm{out}}$, and $\tau_{\mathrm{in}}$, respectively.

### Moments of the occupancy time

First, we consider an individual initially in a state within the target set B. To calculate its occupancy time in B, we follow its pathway, and count the number of visits to target states. The parts of the pathway between two target-state visits are irrelevant for this count. Hence, it is sufficient to follow the pathway of the sub-Markov chain, and count its number of visits to B. That is the number of steps in which the sub-Markov chain is in one of its transient states. Iosifescu (1980) (Theorem 3.2) provides a recursion formula for the moments of this number, which translates here into a formula for the moments of the occupancy time in the target set B for an individual initially in a target state:

(20) $$\tau_{\mathrm{in}}^{1}=N_{S}^{\text{T}}1_{\beta }$$

(21) $$\tau_{\mathrm{in}}^{2}=\left(N_{S}^{\text{T}}-I_{\beta }\right)2\tau_{\mathrm{in}}^{1}+\tau_{\mathrm{in}}^{1}$$

(22) $$=\left(2N_{S}^{\text{T}}-I_{\beta }\right)\tau_{\mathrm{in}}^{1}$$

(23) τink=(NST−Iβ)∑r=1k−1krτinr+τin1, for k≥2,

where $N_{S}={\left(I_{\beta }-U_{S}\right)}^{-1}$ is the fundamental matrix associated with the *sub* Markov chain.

From Eqs. (20), (21), we obtain a formula for the variance of the occupancy time in B for an individual initially in a state within the target set B,

(24) $$\mathrm{Var}\left(\tau_{\mathrm{in}}^{\text{T}}\right)=1_{\beta }^{\text{T}}N_{S}\left(2N_{S}-I_{\beta }\right)-\left(1_{\beta }^{\text{T}}N_{S}\right)\circ \left(1_{\beta }^{\text{T}}N_{S}\right).$$

Second, we consider an individual initially in a non-target state. Since the sub-Markov chain is not defined outside the target states, we cannot directly apply the technique used above. However, we know that, either the individual never enters B, in which case its occupancy time is zero, or it reaches some target state j. In the latter case, the probabilistic fate of the individual after it reaches j is equivalent to the fate of an individual that starts in the target state j; this is the *strong Markov property* (see e.g., Meyn and Tweedie (2009)). Thus, the conditional mean occupancy time, given the first-reached target state j, is the mean occupancy time of an individual initially in j. The unconditional mean occupancy time is the average, over the target states, of the means of the occupancy time of an individual initially in a target state, weighted by the probabilities to reach first these target states. This holds for all moments, and is translated in matrix notation into

(25) $$\tau_{\mathrm{out}}^{k}=A^{\text{T}}\tau_{\mathrm{in}}^{k}$$

where the matrix A contains the probabilities of reaching the target states (defined in Eq. (11)), and the vector $\tau_{\mathrm{in}}^{k}$ is given by Eq. (23). In Appendix A.3, we provide a formal proof of Eq. (25).

From Eq. (25), we obtain a formula for the variance of the occupancy time in B for an individual initially in a non-target state,

(26) $$\mathrm{Var}\left(\tau_{\mathrm{out}}^{\text{T}}\right)=1_{\beta }^{\text{T}}N_{S}\left(2N_{S}-I_{\beta }\right)A-\left(1_{\beta }^{\text{T}}N_{S}A\right)\circ \left(1_{\beta }^{\text{T}}N_{S}A\right),$$

and

(27) $$\mathrm{Var}\left(\tau_{B}^{\text{T}}\right)=\left[\mathrm{Var}\left(\tau_{\mathrm{out}}^{\text{T}}\right):\mathrm{Var}\left(\tau_{\mathrm{in}}^{\text{T}}\right)\right].$$

### Distribution of the occupancy time

First, we calculate the distribution of the occupancy time in B for an individual initially in a state within the target set. Similarly to the moment calculations, this distribution is equivalent to the distribution of the number of steps in which the sub-Markov chain is in one of its transient states. Following Iosifescu (1980) (p. 104), we obtain

(28) $$\left(P\left(\tau_{1}^{\mathrm{in}}=n\right),\ldots ,P\left(\tau_{\beta }^{\mathrm{in}}=n\right)\right)=\left\{\begin{array}{cc} 0_{\beta }^{\text{T}} & \text{ for }n=0\\ 1_{\beta }^{\text{T}}\left(I_{\beta }-U_{S}\right)U_{S}^{n-1} & \text{ for }n\geq 1. \end{array}\right)$$

Second, we calculate the distribution of the occupancy time in B for an individual initially in a non-target state. Similarly to the moment calculations, we use the strong Markov property to deduce this distribution from the distribution of the occupancy time in B for an individual initially in a state within the target set, given in Eq. (28). The probability that the occupancy time is zero, however, requires a special attention. This probability is equal to the probability that the individual dies before it enters any target state; that is one minus the absorbing probability (see Eq. (12)). Hence, the distribution of the occupancy time in B for an individual initially in a non-target state is

(29) $$\left(P\left(\tau_{1}^{\mathrm{out}}=n\right),\ldots ,P\left(\tau_{\alpha }^{\mathrm{out}}=n\right)\right)=\left\{\begin{array}{cc} 1_{\alpha }^{\text{T}}-p_{a}^{\text{T}} & \text{ for }n=0\\ 1_{\beta }^{\text{T}}\left(I_{\beta }-U_{S}\right)U_{S}^{n-1}A & \text{ for }n\geq 1. \end{array}\right)$$

## 5 Correlation between the occupancy times in two sets

Consider two subsets $B_{1}$ and $B_{2}$, of the transient set T. In this section we calculate the correlation between the occupancy time in $B_{1}$ and the occupancy time in $B_{2}$. A positive (resp. negative) correlation means that both of the occupancy times tend to have the same (resp. opposite) “behaviour”, i.e. when one is greater than its mean, the other tends to be greater (resp. smaller) than its mean and vice versa when it is smaller. The correlation between $\tau_{B_{1}}$ and $\tau_{B_{2}}$ is defined as follows

(30) $$\mathrm{Corr}\left(\tau_{B_{1}},\tau_{B_{2}}\right)=\frac{\mathrm{Cov}\left(\tau_{B_{1}},\tau_{B_{2}}\right)}{\sqrt{\mathrm{Var}\left(\tau_{B_{1}}\right)\mathrm{Var}\left(\tau_{B_{2}}\right)}},$$

where the covariance between $\tau_{B_{1}}$ and $\tau_{B_{2}}$ is

(31) $$\mathrm{Cov}\left(\tau_{B_{1}},\tau_{B_{2}}\right)=E\left[\left(\tau_{B_{1}}-\tau_{B_{1}}^{1}\right)\left(\tau_{B_{2}}-\tau_{B_{2}}^{1}\right)\right].$$

The denominator in the right hand side of Eq. (30) is directly calculated with the formulae (24), (26) applied successively to $B_{1}$ and $B_{2}$. To calculate the numerator let us split the set $B_{1}\cup B_{2}$ into three pairwise disjoint sets (possibly empty): the states in $B_{1}$ that are not in $B_{2}$:

(32) $$C_{1}=B_{1}\cap B_{2}^{c},$$

the states in $B_{2}$ that are not in $B_{1}$:

(33) $$C_{2}=B_{2}\cap B_{1}^{c},$$

and the states that are in $B_{1}$ and in $B_{2}$

(34) $$C_{3}=B_{1}\cap B_{2}.$$

Then we have

(35) $$\mathrm{Cov}\left(\tau_{B_{1}},\tau_{B_{2}}\right)=\mathrm{Cov}\left(\tau_{C_{1}}+\tau_{C_{3}},\tau_{C_{2}}+\tau_{C_{3}}\right)$$

(36) $$=\mathrm{Cov}\left(\tau_{C_{1}},\tau_{C_{2}}\right)+\mathrm{Cov}\left(\tau_{C_{1}},\tau_{C_{3}}\right)+\mathrm{Cov}\left(\tau_{C_{2}},\tau_{C_{3}}\right)+\mathrm{Var}\left(\tau_{C_{3}}\right).$$

The covariances in the right hand side of Eq. (36) are calculated with formula (86) applied to the appropriate sets. The variance $\mathrm{Var}\left(\tau_{C_{3}}\right)$ is calculated with formulae (24) and (26) applied to the set $C_{3}$.

If the sets $B_{1}$ and $B_{2}$ are disjoint, Eq. (36) boils down to

(37) $$\mathrm{Cov}\left(\tau_{B_{1}},\tau_{B_{2}}\right)=\frac{1}{2}\left[\mathrm{Var}\left(\tau_{B_{1}\cup B_{2}}\right)\right)-\left(\mathrm{Var}\left(\tau_{B_{1}}\right)-\mathrm{Var}\left(\tau_{B_{2}}\right)\right]$$

(see Appendix A.4 for details). The variances $\mathrm{Var}\left(\tau_{B_{1}\cup B_{2}}\right)$, $\mathrm{Var}\left(\tau_{B_{1}}\right)$, and $\mathrm{Var}\left(\tau_{B_{2}}\right)$ are calculated with the formulae (24), (26) applied to the sets $B_{1}\cup B_{2}$, $B_{1}$, and $B_{2}$, respectively.

### 5.1 Correlation between occupancy time and longevity

In the particular case when the set $B_{2}$ is the *entire* transient set, and $B_{1}=B$ is another target set, the calculations above provide a formula for the correlation between occupancy time in B and the longevity.

Let $\eta_{i}$ denote the longevity for an individual initially in state i. The longevity is the sum of the time spent in all the transient states before death, that is the occupancy time in the entire transient set T (see Caswell (2001)). Therefore the correlation between the occupancy time in the target states and the longevity of an individual can be calculated with the formula (30) applied to $B_{1}=B$ and $B_{2}=T$. In this particular case, the calculations above boil down to

(38) $$\mathrm{Corr}\left(\tau_{B},\eta \right)=\frac{\mathrm{Cov}\left(\tau_{B},\tau_{B^{c}}\right)+\mathrm{Var}\left(\tau_{B}\right)}{\sqrt{\mathrm{Var}\left(\tau_{B}\right)\mathrm{Var}\left(\eta \right)}}.$$

## 6 Behaviour of winners and losers

For want a better term, we call the individuals that eventually reach the target states *winners*. Individuals that never reach the target states are called *losers* (the terminology implies nothing about the desirability of the states). In the Southern Fulmar example with target set $B_{b}$, the winners are individuals that eventually breed, successfully or not, and the losers are individuals that never breed. Two demographic variables are related to the behaviour of the winners: the time required to reach the target set, and the time to return to the target set. In the Southern Fulmar example with target set $B_{b}$, these variables translate into the time to maturation, and the time interval between two breeding attempts. In this Section, we calculate the probability that an individual eventually becomes a winner, and the conditional probability distribution of the time to reach the target set, given that the individual eventually does so. We also provide formulae for the variance and any moments of this distribution. Then, we calculate the probability that a winner returns to the target set, after its first visit. Finally, we calculate the conditional probability distribution of the time to return to the target set, given that the individual eventually returns. We also provide formulae for the variance and any moments of this distribution.

### 6.1 Probability of reaching the target states

The probability that an individual eventually reaches the target set is the probability that the killed Markov chain is absorbed into a target state (see Section 3.1.1). Therefore the probabilities of being a winner, given the current state, are given by the entries of the vector $p_{a}=1^{\text{T}}A$,

(39) $$\mathrm{Pr}\left(\text{individual in }i\text{ becoming a winner }\right)=p_{i}^{a},$$

(40) $$\mathrm{Pr}\left(\text{individual in }i\text{ becoming a loser }\right)=1-p_{i}^{a}.$$

### 6.2 Time to reach the target states

Let $t_{i}^{B}$ denote the random time required to reach the target set B by a winner initially in the non target state i, and

(41) $$t_{B}={\left(t_{1}^{B},\ldots ,t_{\alpha }^{B}\right)}^{\text{T}}.$$

The time required by a winner to reach the target set is the occupancy time of the conditional Markov chain in its *entire* transient set. Indeed, by definition, the conditional Markov chain describes the transitions of winners before they enter the target set. Theorem 3.2 in Iosifescu (1980), applied to the conditional Markov chain, provides a recursion formula for the moments of the occupancy time, which translates here into a formula for the moments of the time required to reach the target states,

(42) tBk=NCT−Iα∑r=1k−1krtBr+tB1,

where $N_{C}={\left(I_{\alpha }-U_{C}\right)}^{-1}$ is the fundamental matrix associated with the conditional Markov chain. In particular, Eq. (42) yields the mean and variance of $t_{B}$,

(43) $$E\left[t_{B}^{\text{T}}\right]=1_{\alpha }^{\text{T}}N_{C}$$

(44) $$\mathrm{Var}\left(t_{B}^{\text{T}}\right)=1_{\alpha }^{\text{T}}N_{C}\left(2N_{C}-I_{\alpha }\right)-\left(1_{\alpha }^{\text{T}}N_{C}\right)\circ \left(1_{\alpha }^{\text{T}}N_{C}\right).$$

Following Iosifescu (1980) (see p. 104), we also obtain a formula for the distribution of $t_{B}$

(45) $$\left(P\left(t_{1}^{B}=n\right),\ldots ,P\left(t_{\alpha }^{B}=n\right)\right)=1_{\alpha }^{\text{T}}\left(I_{\alpha }-U_{C}\right)U_{C}^{n-1}.$$

### 6.3 Return to the target states

A winner will eventually enter the target set but its life may not end there. It may leave B one time-step after its first entrance and never return, it may leave but return eventually, it may stay and leave later, or it may stay forever. An individual returns to the target set if it is in one of the target states at some time, and it visits a target state at a later time, but without passing through any target state in-between.

#### 6.3.1 Return probabilities

The entries of the sub-Markov chain describe the probabilities for an individual to go – in possibly more than one time-step – from one target state to another, without passing through other target states in-between. Hence, the entry $u_{ij}^{S}$ (Eq. (18)) is the probability that an individual in target state α+i returns to B in target state α+j,

(46) $$\mathrm{Pr}\left(\text{individual currently in }i\text{ returns to }B\text{ through }j\right)=u_{ji}^{S}.$$

The probabilities of returning to B regardless in which state are given by the vector

(47) $$p_{r}=U_{S}^{\text{T}}1_{\beta }.$$

#### 6.3.2 Return time

The return time to the target set is the time between two visits to this set. For a demographic Markov chain, it is the time interval between two instances of the demographic events described by the target set, e.g., the number of years between two breeding attempts. This time interval can be as small as 1 if the individual breeds the next year, but it can be infinite if the individual dies before breeding again. To avoid the later scenario, we calculate the conditional probability distribution of the return time, given that the individual does eventually return to B. Caswell (2006) provides formulae for the distribution and the moments of the interval between demographic events that are described by a *single* target state. By using a different method, we generalise this result to events described by several target states.

Let $\mu_{i}$ denote the return time for an individual initially in target state α+i, and

(48) $$\mu ={\left(\mu_{1},\ldots ,\mu_{\beta }\right)}^{\text{T}}.$$

To calculate the probability distribution of μ, we need to calculate the one-time-step transition probabilities from target states to target states, and from target states to non-target states, conditional on eventual return to the target set.

Let $W_{\mathrm{in}}$, of size β×β, denote the matrix which contains the conditional probabilities that an individual initially in a target state moves to a target state in one step, given that it eventually returns to the target set. By definition of conditional probabilities, we have

(49) $$W_{\mathrm{in}}=QD_{r}^{-1}\;\;\text{ of size }\beta \times \beta ,$$

where diagonal of $D_{r}=\text{diag}\;\left(p_{r}\right)$ contains the return probabilities, as defined in Eq. (47), and Q contains the unconditional probabilities that an individual initially in a target state moves to a target state, as defined in Eq. (7). Eq. (49) is derived in detail in Appendix A.5.

Let $W_{\mathrm{out}}$ of size α×β denote the matrix which contains the conditional probabilities that an individual initially in a target state moves to a non-target state, given that it eventually returns to the target set. Similar to the calculation of the transition probabilities of the conditional Markov chain (Section 3.2), the calculation of $W_{\mathrm{out}}$ follows from the definition of conditional probabilities and the Markov properties of the original chain,

(50) $$W_{\mathrm{out}}=D_{a}LD_{r}^{-1}\;\;\text{ of size }\alpha \times \beta ,$$

where $D_{r}=\text{diag}\;\left(p_{r}\right)$ and $D_{a}=\text{diag}\;\left(p_{a}\right)$, respectively, contain on their diagonal the probabilities of return to, and the probabilities of absorption in, the target states, as defined in Eqs. (11), (47). The matrix L contains the unconditional probabilities that an individual initially in a target state moves to a non-target state, as defined in Eq. (7). Eq. (50) is derived in detail in Appendix A.5.

The probability distribution of μ is derived from the matrices $W_{\mathrm{in}}$, $W_{\mathrm{out}}$, and the transition matrix $U_{C}$ of the conditional Markov chain. The return time of an individual initially in the target set is equal to one if its first transition is into the target set. The probabilities of those transitions are obtained by summing the columns of matrix $W_{\mathrm{in}}$, i.e. 

(51) $$\left(P\left(\mu_{1}=1\right),\ldots ,P\left(\mu_{\beta }=1\right)\right)=1_{\beta }^{\text{T}}W_{\mathrm{in}}.$$

To calculate the probabilities that the return time is n, with n≥2, we use the Markov property. An individual with a return time equal to n has first moved to a non-target state, and from there it has reached the target set in n−1 steps. Hence, the probability that the return time is equal to n is the sum, over all non-target states, of the product of the probability that an individual moves to a non-target state and the probability that an individual reaches the target set from this non-target state, in n−1 steps. Hence,

(52) $$\left(P\left(\mu_{1}=n\right),\ldots ,P\left(\mu_{\beta }=n\right)\right)=\left[1_{\alpha }^{\text{T}}\left(I_{\alpha }-U_{C}\right)U_{C}^{n-2}\right]W_{\mathrm{out}},\;\text{ for }n\geq 2.$$

The probabilities that an individual initially in a target state moves to a non-target set are given by the column-sums of the matrix $W_{\mathrm{out}}$. The bracketed term is the probability of reaching the target set in n−1 steps, as given in Eq. (45).

In Appendix A.5, we also derive a formula for the moments of μ. In particular, this formula yields the mean and variance of μ,

(53) $$E\left[\mu \right]=1_{\beta }+W_{\mathrm{out}}^{\text{T}}t_{B}^{1}$$

(54) $$\mathrm{Var}\left(\mu \right)=W_{\mathrm{out}}^{\text{T}}t_{B}^{2}-W_{\mathrm{out}}^{\text{T}}t_{B}^{1}\circ W_{\mathrm{out}}^{\text{T}}t_{B}^{1}.$$

## 7 Example: the Southern Fulmar

The Southern Fulmar is an ice-dependent seabird. This species breeds along the coast of Antarctica and outlying islands, and its individuals forage near the ice edge. Because of its breeding and foraging habitat, the vital rates of the Southern Fulmar are, to some extent, dependent on the sea ice condition. Using the life cycle illustrated in Fig. 1, Jenouvrier et al. (2015) estimated matrix population models for three ice conditions: favourable, ordinary, and unfavourable. From these models, we derive three transient state transition matrices, $U_{\mathrm{fav}},U_{\mathrm{ord}}$, and $U_{\mathrm{unfav}}$, describing transitions and survival in a favourable, ordinary, and unfavourable ice conditions, respectively. These matrices are provided in the Supplementary Material.

We choose to focus on the super-state “breeding attempt”; that is, the target states $B_{b}=\left\{3,4\right\}$. This set plays an important role in the developmental description of an individual. The pre-breeder state describes juvenile individuals, the non-breeder state describes adult individuals that are skipping reproduction, and $B_{b}$ captures the actively reproducing individuals. In this section, we first show the construction of the killed Markov chain, the conditional Markov chain, and the sub Markov chain for $B_{b}$. Then, we illustrate the calculations listed in Table 1, describe their biological meaning for the target set $B_{b}$, and compare them between the three ice conditions. In the Supplementary Material, we provide the matlab code for calculating all the measures presented here. Due to space constraints, we only show matrices associated with the ordinary ice conditions.

### Original Markov chain

The transition probabilities between the transient states of the original Markov chain are extracted from Jenouvrier et al. (2015),

(55) $$U_{\mathrm{ord}}=\left(\begin{array}{cccc} 0.9 & 0 & 0 & 0\\ 0 & 0.63 & 0.07 & 0.18\\ 0.01 & 0.18 & 0.67 & 0.49\\ 0.01 & 0.09 & 0.19 & 0.24 \end{array}\right).$$

The death probability vector m is deduced from $U_{\mathrm{ord}}$,

(56) $$m=1_{4}-U^{\text{T}}1_{4}=\left(\begin{array}{c} 0.08\\ 0.1\\ 0.07\\ 0.09 \end{array}\right).$$

### Constructing the killed Markov chain

The killed chain is the original chain that is stopped as soon as it enters $B_{b}$. The transient states of the killed chain are {1,2}, and the absorbing states are {3,4,d}. Fig. 3 (a) shows the graph of the killed Markov chain. The variables required to describe the transition matrix $P_{K}$ (Eq. (8)) are the matrices $U_{K}$ and K (both extracted from $U_{\mathrm{ord}}$ as shown in Eq. (7)), and the vector $m_{2}$ containing the death probabilities from the non-target states (as defined in Eq. (9)):

$$U_{K}=\left(\begin{array}{cc} 0.9 & 0\\ 0 & 0.63 \end{array}\right),\;\;K=\left(\begin{array}{cc} 0.01 & 0.18\\ 0.01 & 0.09 \end{array}\right),\text{ and }$$

(57) $$m_{2}=\left(\begin{array}{c} 0.08\\ 0.1 \end{array}\right).$$

### Constructing the conditional Markov chain

The conditional Markov chain is the killed chain, conditional on eventual absorption in the target states. The transient states of the conditional chain are {1,2}, and the absorbing state are {3,4}. Fig. 4 (a) shows the graph of the conditional Markov chain. The variables required to construct the transition matrix $P_{C}$ Eq. (8) are the matrices $U_{C}$ and $m_{C}$, both defined in Eq. (14),

(58) $$U_{C}=\left(\begin{array}{cc} 0.9 & 0\\ 0 & 0.63 \end{array}\right),\;\;M_{C}=\left(\begin{array}{cc} 0.061 & 0.25\\ 0.037 & 0.12 \end{array}\right).$$

### Constructing the sub Markov chain

The sub Markov chain is the original chain observed through a filter that makes visible only the target states and the death state. The transient states of the sub chain are the target states {3,4}, and the absorbing state is {d}. Fig. 5 (a) shows the graph of the sub-Markov chain. The variables required to construct the transition matrix $P_{S}$ (Eq. (16)) are the matrix $U_{S}$ (defined in Eq. (18)), and the vector $m_{S}$ (defined in Eq. (17),

(59) $$U_{S}=\left(\begin{array}{cc} 0.7 & 0.58\\ 0.21 & 0.29 \end{array}\right),\;\;m_{S}=\left(\begin{array}{c} 0.09\\ 0.13 \end{array}\right).$$

Now, we illustrate the calculations listed in Table 1 for each ice condition.

### Occupancy time in $B_{b}$.

**Fig. 6:**
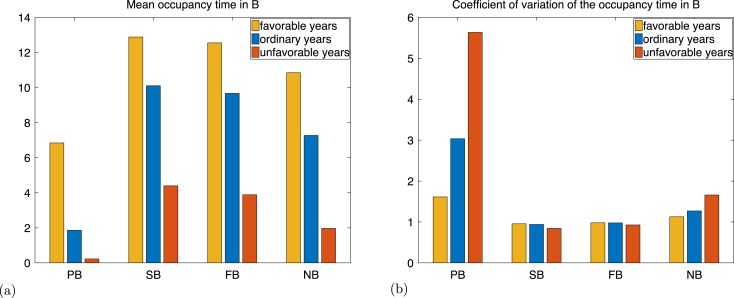
Mean (a) and coefficient of variation (b) of the occupancy time in the set $B_{b}$ for an individual initially in the states pre-breeder (PB), successful breeder (SB), failed breeder (FB), and non breeder (NB).

The occupancy time in the set $B_{b}$ is the *lifetime number of breeding attempts*. Fig. 6 illustrates the mean and coefficient of variation of the lifetime number of breeding attempts (i.e. occupancy time in $B_{b}$), calculated with formulae (20), (24), (25), and (26), applied to $B=B_{b}$.

The mean number of breeding attempt is extremely low under unfavourable ice conditions (Fig. 6). This is a consequence of the long time to maturation under unfavourable ice conditions. In fact, the probability of maturation in unfavourable conditions is only 0.0544. Thus, less than 1/20th of the newborns mature; 19/20 never attempt breeding. However, those that do mature have a relatively large number of breeding attempts (see Fig. 6 (a), initial states successful breeder and failed breeder). This large difference between the mean number of breeding attempt of newborns and adults explains partially the large standard deviation of the number of breeding attempts of newborns, which is almost six times the mean, as shown in Fig. 6 (b). In contrast, under favourable conditions, half of the newborns will reach maturity, which brings the mean number of breeding attempt for newborns closer to the mean number of breeding attempt for adults. As a consequence, the standard deviation of the number of breeding attempts is less than twice its mean.

**Fig. 7:**
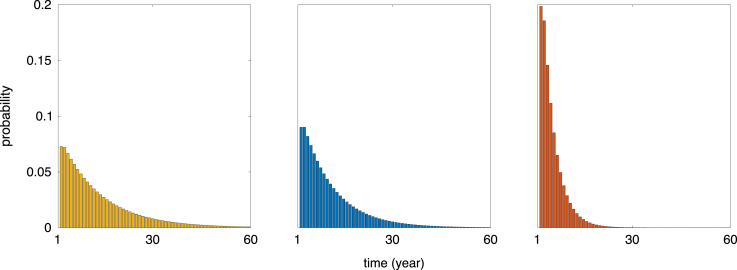
Distributions of the occupancy time in the set $B_{b}$ for an individual starting in the state successful breeder under favourable condition (left), ordinary condition (centre), and unfavourable condition (right).

Fig. 7 illustrates the probability distribution of the lifetime number of breeding attempt for adults under each ice condition, calculated by applying Eq. (28) to the set $B_{b}$. These distributions are approximately geometric, with parameter 1−p, where $p=\frac{1}{2}\left(p_{1}^{r}+p_{2}^{r}\right)$ is the average probability of returning to $B_{b}$ (see Eq. (47)), which ranges from 0.9272 (favourable conditions) to 0.6951 (unfavourable conditions). The mean and coefficient of variation of this distribution are $\frac{1}{1-p}$ and $\sqrt{p}$, respectively. This explains why the relative decrease of the mean number of breeding events after maturation between favourable ice conditions and unfavourable conditions is approximately 0.7, but the relative decrease of the coefficient of variation is only 0.1, as shown in Fig. 7.

### Correlation between occupancy time and longevity.

**Fig. 8:**
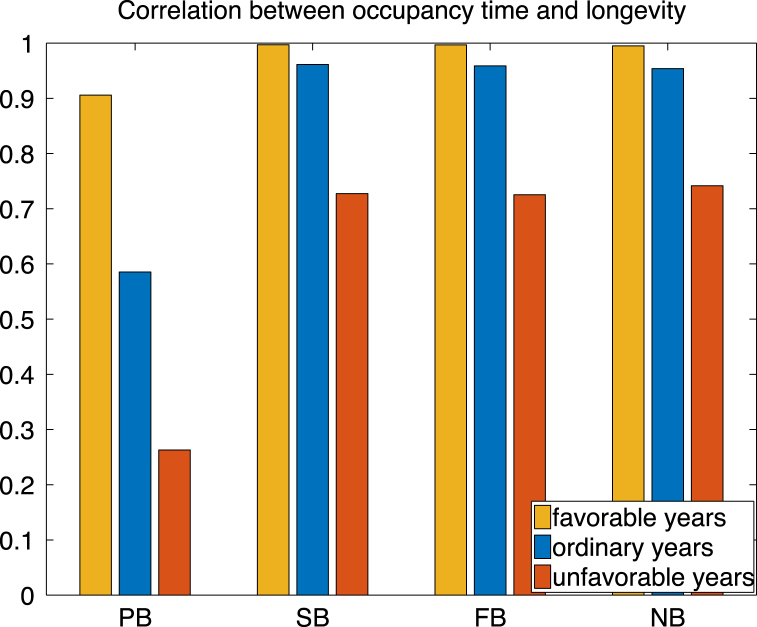
Correlation between occupancy time in $B_{b}$ and the expected longevity for an individual initially in the states pre-breeder (PB), successful breeder (SB), failed breeder (FB), and non breeder (NB).

The correlation between the longevity of an individual and its number of breeding attempts provides information on how longevity affects the total number of breeding attempts. Using formula (38), we find a positive correlation (Fig. 8) for each ice condition. Indeed, we expect that on average a particularly large longevity results in a particularly large number of breeding attempt and vice versa. Because newborn individual cannot breed before maturity, the correlation between longevity and breeding attempts for newborn is smaller than the correlation for adults. Under favourable ice conditions, individuals spend most of their lives in $B_{b}$, and the correlation approaches 1.

### Time required to reach $B_{b}$.

**Fig. 9:**
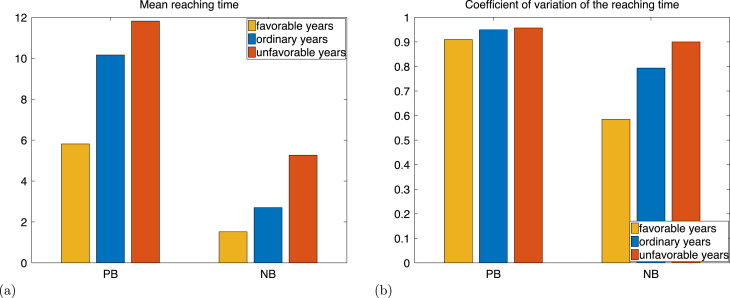
Mean (a) and coefficient of variation (b) of the time to reach the set $B_{b}$ for individual initially in the states pre-breeder (PB), and non breeder (NB).

The target states in $B_{b}$ describe active reproduction, and the time to reach $B_{b}$ is the *time to maturation*. Fig. 9 illustrates the mean and coefficient of variation of this time, calculated with formulae (43), (44) applied to $B=B_{b}$. The average maturation time under unfavourable ice conditions among individuals that do successfully mature is twice as large as that under favourable ice conditions.

### Time required to return to $B_{b}$.

**Fig. 10:**
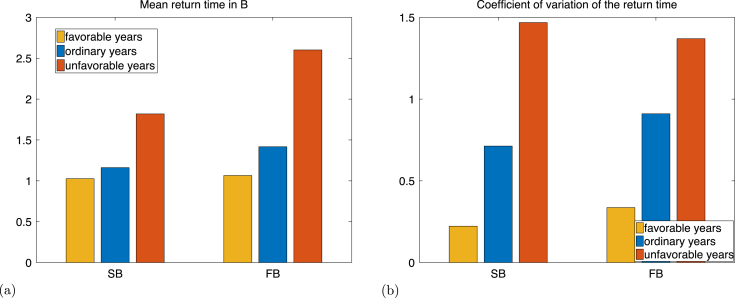
Mean (a) and coefficient of variation (b) of the time to return to the set $B_{b}$ for an individual initially in the states successful breeder (SB), and failed breeder (FB).

This time corresponds to the time between two breeding attempts, which is a measure of inter-birth interval and an indicator of the individual’s reproduction consistency. This time is 1 for individuals that never skip reproduction, and is infinite for individuals that reproduce only once. In terms of the Markov chain, this time is the return time to the set $B_{b}$. The mean return time increases as the ice condition becomes less favourable (Fig. 10). Unlike for the maturation time, the coefficient of variation of the time between breeding increases significantly as well. One reason is that non-breeding individuals have a smaller probability of attempting breeding the following year under unfavourable ice conditions ($p_{2}^{a}=0.19$) than under favourable ice conditions ($p_{2}^{a}=0.66$). This results in more variation in the distribution of the time required to attempt breeding after a non breeding season under unfavourable ice conditions than under favourable ice conditions, as shown in Figures S1 and S2 provided in the Supplementary Material.

## 8 Discussion

From birth to death, an individual goes through a sequence of random events (e.g., surviving, developing, mating, reproducing, growing, dispersing, moving among social or occupational classes, or changing health status). This results in a stochastic pathway through the individual state space. Many important life history traits are direct functions of these pathways (e.g., longevity, reproductive output, age at maturity) and are often related to the concept of occupancy time in a state. Absorbing Markov chains are a powerful model framework to analyse these stochastic pathways, but current theory can only calculate the occupancy times in a *single* state. However, often the occupancy time in a *set* of states is desired. For example, a size range in a size-classified model, an age class in a age×stage model, or a set of locations in a spatial model are all sets of states. We have presented a new mathematical approach to absorbing Markov chains that generalises the occupancy calculations to sets of states and enlarges the list of demographic measures that can be calculated from a demographic model.

For any number of target states, we provide formulae to calculate any moments (e.g., mean and variance) and the probability distribution of

• the occupancy time in a target set
• the time required to reach a target set
• the time required to return to a target set.

Last but not least, we provide a formula to calculate the correlation between the occupancy times in two different sets. These formulae are straightforward to calculate in matrix oriented software.

In demographic models based on absorbing Markov chains, any life history trait that is a function of the individual’s pathway is stochastic. The calculation of the resulting distribution of this trait among individuals is complicated, and is often approximated by its mean and, sometime, its variance. While the current theory can calculate any moment of the occupancy time in a single state, our generalisation to the set occupancy time provides also a formula for its probability distribution.

As the questions in population ecology become more sophisticated and data become more detailed, the use of multistate and hyperstate matrix models will become increasingly important (Roth and Caswell, 2016). For these models, it is often useful to consider marginal sets. For example, in a age×stage-structured model, the marginal set age x describes individuals in age class x independently of their stage. Similarly, an increasingly number of studies use integral projection models (Ellner et al., 2016) which are approximated – and then analysed – by matrix projection models with highly detailed i-state space (e.g., large number of size classes). For these models, the occupancy in a single state is often of no interest, while the occupancy in a set of state can be biological important (e.g., range of ages or sizes corresponding to a specific developmental stages). Our work generalises the occupancy calculations for these two highly powerful family of models.

In human demography, multistate models often combine age classes with health or social categories. Studies may focus on the *expected occupancy times* (e.g., Siegel (2012) and Clark et al. (2016)), but very little use is made of the Markov chain framework presented here. Applying our results to such human demographic models would significantly improve the calculations of the occupancy by providing the variance and its probability distribution.

The only data required for our calculations are the transition probabilities between the states that describe the individuals. These probabilities can be obtained from most population projection matrices. The availability of projection matrix data has been improved by the databases COMPADRE for plant and COMADRE for animals [21], [22]. Hence, our approach to absorbing Markov chain makes the analysis of occupancy times accessible for a wide range of existing projection matrix models.

The power of our results is to provide easy-to-use formulae to calculate demographic measures that seem, a priori, to be complicated. All the indices listed in Table 1 can be calculated with matrix expressions, which are very easy to implement in matrix oriented softwares. In the Supplementary Material, we provide the matlab code that calculates the results listed in Table 1 from a given transition matrix U and a set of transient states B. We also provide the matlab code for calculating the results of the Southern Fulmar example presented in Section 7.

In ecology and in human demography, discrete time models are widely used for their simplicity in their formulation and for their fit with data observed at specific times. However, the underlying processes of these dynamics are certainly continuous in time. Hence, extending the occupancy calculations to continuous time Markov chains would significantly enlarge the range of applications.

## Appendix A

To derive our formulae, we need to define formally the stochastic process described by the transition matrices $P,P_{K},P_{C},$ and $P_{S}$. The Markov chain associated with the transition matrix P describes a stochastic process ${\left\{X_{t}\right\}}_{t\geq 0}$ taking values in the i-state space S and satisfying the Markov property

(60) $$P\left(X_{t+1}=i|X_{t}=j,X_{t-1}=j_{t-1},\ldots ,X_{0}=j_{0}\right)=p_{ji}$$

for any $i,j,j_{0},\ldots ,j_{t-1}\in S$. Likewise, the killed Markov chain, the conditional Markov chain, and the sub-Markov chain describe the stochastic processes ${\left\{X_{t}^{K}\right\}}_{t\geq 0}$ ${\left\{X_{t}^{C}\right\}}_{t\geq 0}$ ${\left\{X_{t}^{B}\right\}}_{t\geq 0}$, which satisfy the Markov property, respectively.

### A.1 Derivation of the matrix $U_{S}$ and the vector $m_{S}$

Let i,j be two target states. The entry $u_{j-\alpha ,i-\alpha }^{S}$ is the probability that an individual initially in target state j to reach the state i without passing by any other states in B. Define the stopping time $T=\min\left\{t\geq 1|X_{t}\in B\right\}$. The time T is the random time – possibly infinite – at which the individual will enter the set B. In particular, $X_{T}$ is the state through which the individual enters for its first time in B. Then we can rewrite $u_{j-\alpha ,i-\alpha }^{S}$ as

(61) $$u_{j-\alpha ,i-\alpha }^{S}=P_{i}\left(X_{T}=j\right).$$

By definition of the absorbing probabilities (Eq. (11)), for a non target state $k\in B^{c}$, we have

(62) $$a_{j-\alpha ,k}=P_{k}\left(X_{T}=j\right).$$

Using the Chapman–Kolmogorov equation (see e.g., Meyn and Tweedie (2009)), we obtain

(63) $$P_{i}\left(X_{T}=j\right)=P_{i}\left(X_{1}=j\right)+\underset{k\in B^{c}}{\sum }P_{i}\left(X_{T}=j|X_{1}=k\right)P_{i}\left(X_{1}=k\right)=P_{i}\left(X_{1}=j\right)+\underset{k\in B^{c}}{\sum }P_{k}\left(X_{T}=j\right)P_{i}\left(X_{1}=k\right)=P_{i}\left(X_{1}=j\right)+\underset{k\in B^{c}}{\sum }a_{\alpha -j,k}P_{i}\left(X_{1}=k\right)=u_{ji}+\underset{k\in B^{c}}{\sum }a_{\alpha -j,k}u_{ki}.$$

In matrix notation, equation (63) is equivalent to Eq. (18), i.e. 

(64) $$U_{S}=AL+Q$$

where the matrices L and Q are extracted from the matrix U, as in Eq. (7).

### A.2 Proof that $X_{C}$ is a Markov chain

Iosifescu (1980) (section 3.2.9) proves that an absorbing Markov chain, with respect to the conditional probability that it is absorbed by a specific state, is still an absorbing Markov chain. Here, we generalise this statement to the condition that the chain is absorbed in a specific set of states.

Let us define the event $A=\left\{X^{K}\text{ is absorbed in the target set }B\right\}$, i.e. the killed chain is absorbed in the target set. We consider the stochastic process $X^{C}$, living on the space T, defined by

(65) $$P\left(X_{t}^{C}\in B\right)=P\left(X_{t}^{K}\in B|A\right)$$

for any measurable set B⊂S. To ease the notation, we write $P\left(X_{t}^{K}\in B|A\right)=P_{A}\left(X_{t}^{K}\in B\right)$.

By definition, the process $X^{C}$ corresponds to the killed Markov chain, where trajectories encountering death before target states are set aside. We first prove that $X^{C}$ is a Markov chain and then we show that its transition probabilities are describe by the matrix $P_{C}$ defined in Section 3.2. As a consequence, this proves that the conditional Markov chain is indeed a Markov chain and that it corresponds to the killed Markov chain, where trajectories encountering death before target states are set aside.

To prove that $X^{C}$ is a Markov chain, we only need to show that it satisfies the Markov property, i.e. 

(66) $$P\left(X_{t+1}^{C}=i_{t+1}|X_{t}^{C}=i_{t},\ldots ,X_{0}^{C}=i_{0}\right)=P\left(X_{t+1}^{C}=i_{t+1}|X_{t}^{C}=i_{t}\right),$$

for $\left(i_{0},\ldots ,i_{t+1}\right)\in T^{t+2}$.

Fix $\left(i_{0},\ldots ,i_{t+1}\right)\in T^{t+2}$, and define the event $B_{s}=\left\{X_{s}^{K}=i_{s},\ldots ,X_{t}^{K}=i_{0}\right\}$, for 0≤s≤t.

From the definitions of conditional probabilities and the process $X^{C}$, we have

(67) $$P\left(X_{t+1}^{C}=i_{t+1}|X_{t}^{C}=i_{t},\ldots ,X_{0}^{C}=i_{0}\right)=\frac{P\left(\left\{X_{t+1}^{K}=i_{t+1}\right\}\cap B_{0}\cap A\right)}{P\left(A\cap B_{0}\right)}$$

If $\left(i_{0},\ldots ,i_{t}\right)\notin B^{t+1}$, then

$$P\left(A\cap B_{s}\right)=P\left(A|B_{s}\right)P\left(B_{s}\right)=P\left(A|X_{t}^{K}=i_{t}\right)P\left(B_{s}\right)p_{i_{t}}^{a}P\left(B_{s}\right),$$

for any 0≤s≤t. The second equality is a consequence of the Markov property of the killed Markov chain, and the third equality follows from the definition of the absorbing probability vector $p_{a}$ (see Eq. (12)).

Similarly,

(68) $$P\left(\left\{X_{t+1}^{K}=i_{t+1}\right\}\cap B_{s}\cap A\right)=P\left(\left\{X_{t+1}^{K}=i_{t+1}\right\}\cap A|B_{s}\right)P\left(B_{s}\right)$$

(69) $$=P\left(\left\{X_{t+1}^{K}=i_{t+1}\right\}\cap A|X_{t}^{K}=i_{t}\right)P\left(B_{s}\right)$$

(70) $$=P\left(B_{s}\right)I_{B}\left(i_{t+1}\right)P_{i_{t}}\left(X_{t+1}^{K}=i_{t+1}\right)$$

(71) $$+P\left(B_{s}\right)\left(1-I_{B}\left(i_{t+1}\right)\right)P\left(A|X_{t+1}^{K}=i_{t+1}\right)P_{i_{t}}\left(X_{t+1}^{K}=i_{t+1}\right)$$

(72) $$=P\left(B_{s}\right)\left[I_{B}\left(i_{t+1}\right)P_{i_{t}}\left(X_{t+1}^{K}=i_{t+1}\right)\right)+\left(\left(1-I_{B}\left(i_{t+1}\right)\right)p_{i_{t+1}}^{a}P_{i_{t}}\left(X_{t+1}^{K}=i_{t+1}\right)\right]$$

where $I_{B}\left(k\right)$ equals 1 if k∈B and 0 otherwise.

If $i_{t}\in B$, then $B_{s}\subset A$, for 0≤s≤t, and

(73) $$\frac{P\left(\left\{X_{t+1}^{K}=i_{t+1}\right\}\cap B_{s}\cap A\right)}{P\left(A\cap B_{s}\right)}=\frac{P\left(\left\{X_{t+1}^{K}=i_{t+1}\right\}\cap B_{s}\right)}{P\left(B_{s}\right)}$$

(74) $$=P\left(X_{t+1}^{K}=i_{t+1}|B_{s}\right)$$

(75) $$=P\left(X_{t+1}^{K}=i_{t+1}|X_{t}^{K}=i_{t}\right).$$

Eqs. (72), (75) imply that the ratio on the right hand side of Eq. (67) does not depend on s, for 0≤s≤t. In particular,

(76) $$P\left(X_{t+1}^{C}=i_{t+1}|X_{t}^{C}=i_{t},\ldots ,X_{0}^{C}=i_{0}\right)=\frac{P\left(\left\{X_{t+1}^{K}=i_{t+1}\right\}\cap B_{t}\cap A\right)}{P\left(A\cap B_{t}\right)}$$

(77) $$=P\left(X_{t+1}^{C}=i_{t+1}|X_{t}^{C}=i_{t}\right).$$

This prove that $X^{C}$ satisfies the Markov property.

The transition probabilities of the Markov chain $X^{C}$ follow from the equations above. For j∈T and i∉B,

(78) $$P\left(X_{t+1}^{C}=j|X_{t}^{C}=i\right)=\frac{p_{j}^{a}p_{ji}^{K}}{p_{i}^{a}},$$

with the convention that $p_{k}^{a}=1$ for k∈B. And we have for i,j∈B,

(79) $$P\left(X_{t+1}^{C}=j|X_{t}^{C}=i\right)=1.$$

It follows form Eqs. (78), (79) that the transition probabilities of the Markov chain $X^{C}$ are given by the matrix $P_{C}$ defined in Section 3.2, i.e. 

(80)

where

(81) $$U_{C}=D_{a}U_{K}D_{a}^{-1}\;\text{ and }\;M_{C}=KD_{a}^{-1},$$

where $D_{a}=\text{diag}\;\left(p_{a}\right)$ is a diagonal matrix with, on the diagonal, the probabilities of absorption in the target states.

### A.3 Moments of occupancy times

To calculate the moments of the occupancy time for individuals initially outside the target set B, we use the strong Markov property. If the individual never enters in B, then its occupancy time is zero. If it does enter in B, say through the state j, then the law of its occupancy time is equal to the law of the occupancy time for an individual starting in the state j. To fix the idea, consider the state $i\in B^{c}$. Then

(82) $$E\left(\tau_{i}^{m}\right)=\overset{\infty }{\underset{\ell =1}{\sum }}\ell^{m}P\left(\tau_{i}=\ell \right)=\overset{\infty }{\underset{\ell =1}{\sum }}\ell^{m}\left(\underset{j\in B}{\sum }P\left(\tau_{i}=\ell |\text{enter in }B\text{ in }j\right)\right)\left(P\left(\text{enter in }B\text{ in }j\right)\right)=\overset{\infty }{\underset{\ell =1}{\sum }}\ell^{m}\left(\underset{j\in B}{\sum }P\left(\tau_{j}=\ell \right)a_{ji}\right)=\underset{j\in B}{\sum }a_{ji}\overset{\infty }{\underset{\ell =1}{\sum }}\ell^{m}P\left(\tau_{j}=\ell \right)=\underset{j\in B}{\sum }a_{ji}E\left(\tau_{j}^{m}\right),$$

where $a_{ji}$ is the probability that the killed Markov chain, starting in state i, is absorbed by the state j, as in Section 3.1.1. In matrix notation, Eq. (82) is equivalent to

(83) $$\tau_{\mathrm{out}}^{k}=A^{\text{T}}\tau_{\mathrm{in}}^{k}.$$

### A.4 Covariance between the occupancy times in two disjoint sets

Here, we calculate the covariance between the occupancy time in two disjoint subsets $B_{1}$ and $B_{2}$, of the transient set T. As stated in the main text, the covariance between $\tau_{B_{1}}$ and $\tau_{B_{2}}$ is

(84) $$\mathrm{Cov}\left(\tau_{B_{1}},\tau_{B_{2}}\right)=E\left[\left(\tau_{B_{1}}-\tau_{B_{1}}^{1}\right)\left(\tau_{B_{2}}-\tau_{B_{2}}^{1}\right)\right].$$

We rewrite the covariance between $\tau_{B_{1}}$ and $\tau_{B_{2}}$ in terms of their variances and the variance of their sum,

(85) $$\mathrm{Cov}\left(\tau_{B_{1}},\tau_{B_{2}}\right)=\frac{1}{2}\left[\mathrm{Var}\left(\tau_{B_{1}}+\tau_{B_{2}}\right)\right)-\left(\mathrm{Var}\left(\tau_{B_{1}}\right)-\mathrm{Var}\left(\tau_{B_{2}}\right)\right].$$

Since the sets $B_{1}$ and $B_{2}$ are disjoint, the occupancy time in the union $B_{1}\cup B_{2}$ is the sum of the occupancy times in each of the subsets. Thus,

(86) $$\mathrm{Cov}\left(\tau_{B_{1}},\tau_{B_{2}}\right)=\frac{1}{2}\left[\mathrm{Var}\left(\tau_{B_{1}\cup B_{2}}\right)-\mathrm{Var}\left(\tau_{B_{1}}\right)\right)-\left(\mathrm{Var}\left(\tau_{B_{2}}\right)\right].$$

The variances $\mathrm{Var}\left(\tau_{B_{1}\cup B_{2}}\right)$, $\mathrm{Var}\left(\tau_{B_{1}}\right)$, and $\mathrm{Var}\left(\tau_{B_{2}}\right)$ are calculated with the formulae (24), (26) applied to the sets $B_{1}\cup B_{2}$, $B_{1}$, and $B_{2}$, respectively.

### A.5 One-step transition probabilities from B given return

Let $w_{ij}^{\mathrm{in}}$ be the conditional probability that an individual in target state α+j moves to the target state α+i, in one time-step, given that it eventually returns to the target set. Then,

(87) $$w_{ji}^{\mathrm{in}}≔P_{\alpha +i}\left(X_{1}=\alpha +j|T<\infty \right)=\frac{P_{\alpha +i}\left(X_{1}=\alpha +j,T<\infty \right)}{P_{\alpha +i}\left(T<\infty \right)}=\frac{P_{\alpha +i}\left(X_{1}=\alpha +j\right)}{P_{\alpha +i}\left(T<\infty \right)}=\frac{u_{\alpha +j,\alpha +i}}{\underset{\ell \in B}{\sum }u_{\ell i}^{B}}=\frac{u_{\alpha +j,\alpha +i}}{p_{i}^{r}},$$

where $p_{r}$ describes the return probabilities, as defined in Eq. (47). Thus,

(88) $$W_{\mathrm{in}}=QD_{r}^{-1}\;\;\text{ of size }\beta \times \beta ,$$

where $D_{r}=\text{diag}\;\left(p_{r}\right)$ and the matrix Q is extracted from the matrix U, as in Eq. (7). Let $w_{ij}^{\mathrm{out}}$ be the conditional probability that an individual in target state α+j moves to the non-target state i, in one time-step, given that it eventually returns to the target set. Then

(89) $$w_{ji}^{\mathrm{out}}≔P_{\alpha +i}\left(X_{1}=j|T<\infty \right)=\frac{P_{\alpha +i}\left(T<\infty |X_{1}=j\right)P_{\alpha +i}\left(X_{1}=j\right)}{P_{\alpha +i}\left(T<\infty \right)}=\frac{P_{\alpha +i}\left(X_{1}=j\right)P_{\alpha +j}\left(T<\infty \right)}{P_{\alpha +i}\left(T<\infty \right)}=\frac{u_{j,\alpha +i}\underset{\ell \in B}{\sum }a_{\ell j}}{\underset{\ell \in B}{\sum }u_{\ell i}^{B}}=\frac{u_{j,\alpha +i}p_{j}^{a}}{p_{i}^{r}},$$

where the vector $p_{a}$ describes the probabilities of absorption in the target states, as defined in Eq. (11). Thus,

(90) $$W_{\mathrm{out}}=D_{a}LD_{r}^{-1}\;\;\text{ of size }\alpha \times \beta ,$$

where $D_{r}=\text{diag}\;\left(p_{r}\right)$, $D_{a}=\text{diag}\;\left(p_{a}\right)$, and the matrix L is extracted form the matrix U, as in Eq. (7).

Now, we derive the moments of μ, conditional on the individual returning to the target set. Let α+i be a target state. Then

(91) $$E\left[\mu_{i}^{k}|T<\infty \right]=\overset{\infty }{\underset{n=1}{\sum }}n^{k}P\left(\mu_{i}=n|T<\infty \right)$$

(92) $$=P\left(\mu_{i}=1|T<\infty \right)+\overset{\infty }{\underset{n=2}{\sum }}n^{k}\underset{j\in B^{c}}{\sum }P\left(t_{j}^{B}=n-1\right)P_{i}\left(X_{1}=j|T<\infty \right)$$

(93) $$=P\left(\mu_{i}=1|T<\infty \right)+\underset{j\in B^{c}}{\sum }P_{i}\left(X_{1}=j|T<\infty \right)\overset{\infty }{\underset{n=1}{\sum }}{\left(n+1\right)}^{k}P\left(t_{j}^{B}=n\right)$$

(94) =P(μi=1|T<∞)+∑j∈BcPi(X1=j|T<∞)∑n=1∞∑r=0kkrnrP(tjB=n)

(95) =P(μi=1|T<∞)+∑j∈BcPi(X1=j|T<∞)∑r=0kkrtBjr

(96) =1+∑j∈Bcwjiout∑r=1kkrtBjr.

Hence, in matrix notation,

(97) E[μk|T<∞]=1β+∑r=1kkrWoutTtBr.

## Appendix B Supplementary data

MMC S1 Figures S1 and S2. S1 shows the distribution of the time required to return to the set target set B_b_ for an individual initially in the state successful breeder, under each environmental conditions. S2 shows the distribution of the time required to reach the set B_b_ for an individual initially in the state non breeder, under each environmental conditions.

MMC S2 This file contains the MATLAB codes to carry out the calculations presented in this paper for any transition matrix and any target set. This file contains also the transition matrices used in the Example.
