# Supplementary material for: Occupancy time in sets of states for demographic models
Source: Theor Popul Biol. 2018 Mar;120:62–77. doi: 10.1016/j.tpb.2017.12.007 (PMC5861321; doi:10.1016/j.tpb.2017.12.007)
Supplement: MMC S1 — Figures S1 and S2. S1 shows the distribution of the time required to return to the set target set Bb for an individual initially in the state successful breeder, under each environmental conditions. S2 shows the distribution of the time required to reach the set Bb for an individual initially in the state non breeder, under each environmental conditions. [file mmc1.pdf]

A Appendices

To derive our formulae, we need to define formally the stochastic process described by the transition matrices $\mathbf{P}, \mathbf{P}_{\mathbb{K}}, \mathbf{P}_{\mathbb{C}}$, and $\mathbf{P}_{\mathbb{S}}$. The Markov chain associated with the transition matrix $\mathbf{P}$ describes a stochastic process $\{\mathbf{X}_t\}_{t\geq 0}$ taking values in the i-state space $\mathcal{S}$ and satisfying the Markov property

$$\mathbb{P}\left(\mathbf{X}_{t+1} = i|\mathbf{X}_t = j, X_{t-1} = j_{t-1}, \dots, \mathbf{X}_0 = j_0\right) = p_{ji} \tag{60}$$

for any $i, j, j_0, \dots, j_{t-1} \in \mathcal{S}$. Likewise, the killed Markov chain, the conditional Markov chain, and the sub-Markov chain describe the stochastic processes $\{\mathbf{X}_t^{\mathbb{K}}\}_{t\geq 0}$ $\{\mathbf{X}_t^{\mathbb{C}}\}_{t\geq 0}$ $\{\mathbf{X}_t^{\mathcal{B}}\}_{t\geq 0}$, which satisfy the Markov property, respectively.

A.1 Derivation of the matrix $\mathbf{U}_{\mathbb{S}}$ and the vector $\mathbf{m}_{\mathbb{S}}$

Let i, j be two target states. The entry $u^{\mathbb{S}}_{j-\alpha,i-\alpha}$ is the probability that an individual initially in target state j to reach the state i without passing by any other states in $\mathcal{B}$. Define the stopping time $T = \min\{t \geq 1|\mathbf{X}_t \in \mathcal{B}\}$. The time T is the random time — possibly infinite — at which the individual will enter the set $\mathcal{B}$. In particular, $\mathbf{X}_T$ is the state through which the individual enters for its first time in $\mathcal{B}$. Then we can rewrite $u^{\mathbb{S}}_{j-\alpha,i-\alpha}$ as

$$u^{\mathbb{S}}_{j-\alpha,i-\alpha} = \mathbb{P}_i\left(\mathbf{X}_T = j\right). \tag{61}$$

By definition of the absorbing probabilities (eqn. 11), for a non target state $k \in \mathcal{B}^c$, we have

$$a_{j-\alpha,k} = \mathbb{P}_k\left(\mathbf{X}_T = j\right). \tag{62}$$

Using the Chapman-Kolmogorov equation (see e.g., Meyn and Tweedie [2009]), we obtain

$$\begin{aligned}
\mathbb{P}_i\left(\mathbf{X}_T = j\right) &= \mathbb{P}_i\left(\mathbf{X}_1 = j\right) + \sum_{k\in\mathcal{B}^c} \mathbb{P}_i\left(\mathbf{X}_T = j|\mathbf{X}_1 = k\right)\mathbb{P}_i\left(\mathbf{X}_1 = k\right) \\
&= \mathbb{P}_i\left(\mathbf{X}_1 = j\right) + \sum_{k\in\mathcal{B}^c} \mathbb{P}_k\left(\mathbf{X}_T = j\right)\mathbb{P}_i\left(\mathbf{X}_1 = k\right) \\
&= \mathbb{P}_i\left(\mathbf{X}_1 = j\right) + \sum_{k\in\mathcal{B}^c} a_{\alpha-j,k}\mathbb{P}_i\left(\mathbf{X}_1 = k\right) \\
&= u_{ji} + \sum_{k\in\mathcal{B}^c} a_{\alpha-j,k}u_{ki}.
\end{aligned} \tag{63}$$

In matrix notation, equation (63) is equivalent to equation (18), i.e.

$$\mathbf{U}_{\mathbb{S}} = \mathbf{A}\mathbf{L} + \mathbf{Q} \tag{64}$$

where the matrices $\mathbf{L}$ and $\mathbf{Q}$ are extracted from the matrix $\mathbf{U}$, as in equation (7).

A.2 Proof that $\mathrm{X}_{\mathbb{C}}$ is a Markov chain

Iosifescu [1980] (section 3.2.9) proves that an absorbing Markov chain, with respect to the conditional probability that it is absorbed by a specific state, is still an absorbing Markov chain. Here, we generalise this statement to the condition that the chain is absorbed in a specific set of states.

Let's define the event $A = \{\mathbf{X}^{\mathbb{K}}$ is absorbed in the target set $\mathcal{B}\}$, i.e. the killed chain is absorbed in the target set. We consider the stochastic process $\mathbf{X}^{\mathbb{C}}$, living on the space $\mathcal{T}$, defined by

$$\mathbb{P}\left(\mathbf{X}_t^{\mathbb{C}} \in B\right) = \mathbb{P}\left(\mathbf{X}_t^{\mathbb{K}} \in B|A\right) \tag{65}$$

for any measurable set $B \subset \mathcal{S}$. To ease the notation, we write $\mathbb{P}\left(\mathbf{X}_t^{\mathbb{K}} \in B|A\right) = \mathbb{P}_A\left(\mathbf{X}_t^{\mathbb{K}} \in B\right)$.

By definition, the process $\mathbf{X}^{\mathbb{C}}$ corresponds to the killed Markov chain, where trajectories encountering death before target states are set aside. We first prove that $\mathbf{X}^{\mathbb{C}}$ is a Markov chain and then we show that its transition probabilities are describe by the matrix $\mathbf{P}_{\mathbb{C}}$ defined in Section 3.2. As a consequence, this proves that the conditional Markov chain is indeed a Markov chain and that it corresponds to the killed Markov chain, where trajectories encountering death before target states are set aside.

To prove that $\mathbf{X}^{\mathbb{C}}$ is a Markov chain, we only need to show that it satisfies the Markov property, i.e.

$$\mathbb{P}\left(\mathbf{X}_{t+1}^{\mathbb{C}} = i_{t+1}|\mathbf{X}_t^{\mathbb{C}} = i_t, \dots, \mathbf{X}_0^{\mathbb{C}} = i_0\right) = \mathbb{P}\left(\mathbf{X}_{t+1}^{\mathbb{C}} = i_{t+1}|\mathbf{X}_t^{\mathbb{C}} = i_t\right), \tag{66}$$

for $(i_0, \dots, i_{t+1}) \in \mathcal{T}^{t+2}$.

Fix $(i_0, \dots, i_{t+1}) \in \mathcal{T}^{t+2}$, and define the event $B_s = \{\mathbf{X}_s^{\mathbb{K}} = i_s, \dots, \mathbf{X}_t^{\mathbb{K}} = i_0\}$, for $0 \le s \le t$.

From the definitions of conditional probabilities and the process $\mathbf{X}^{\mathbb{C}}$, we have

$$\mathbb{P}\left(\mathbf{X}_{t+1}^{\mathbb{C}} = i_{t+1}|\mathbf{X}_t^{\mathbb{C}} = i_t, \dots, \mathbf{X}_0^{\mathbb{C}} = i_0\right) = \frac{\mathbb{P}\left(\{\mathbf{X}_{t+1}^{\mathbb{K}} = i_{t+1}\} \cap B_0 \cap A\right)}{\mathbb{P}\left(A \cap B_0\right)} \tag{67}$$

If $(i_0, \ldots, i_t) \notin \mathcal{B}^{t+1}$, then

$$\begin{aligned}\mathbb{P}(A \cap B_s) &= \mathbb{P}(A|B_s)\,\mathbb{P}(B_s) \\ &= \mathbb{P}\left(A|\mathbf{X}^{\mathbb{K}}_t = i_t\right)\mathbb{P}(B_s) \\ p^a_{i_t}\mathbb{P}(B_s)\,, \end{aligned}$$

for any $0 \leq s \leq t$. The second equality is is a consequence of the Markov property of the killed Markov chain, and the third equality follows from the definition of the absorbing probability vector $\mathbf{p}_a$ (see eqn 12).

Similarly,

$$\begin{aligned}\mathbb{P}\left(\{\mathbf{X}^{\mathbb{K}}_{t+1} = i_{t+1}\} \cap B_s \cap A\right) &= \mathbb{P}\left(\{\mathbf{X}^{\mathbb{K}}_{t+1} = i_{t+1}\} \cap A|B_s\right)\mathbb{P}(B_s) && (68)\\ &= \mathbb{P}\left(\{\mathbf{X}^{\mathbb{K}}_{t+1} = i_{t+1}\} \cap A|\mathbf{X}^{\mathbb{K}}_t = i_t\right)\mathbb{P}(B_s) && (69)\\ &= \mathbb{P}(B_s)\,\mathbb{I}_{\mathcal{B}}(i_{t+1})\mathbb{P}_{i_t}\left(\mathbf{X}^{\mathbb{K}}_{t+1} = i_{t+1}\right) && (70)\\ &\quad + \mathbb{P}(B_s)\,(1-\mathbb{I}_{\mathcal{B}}(i_{t+1}))\mathbb{P}\left(A|\mathbf{X}^{\mathbb{K}}_{t+1} = i_{t+1}\right)\mathbb{P}_{i_t}\left(\mathbf{X}^{\mathbb{K}}_{t+1} = i_{t+1}\right) && (71)\\ &= \mathbb{P}(B_s)\left[\mathbb{I}_{\mathcal{B}}(i_{t+1})\mathbb{P}_{i_t}\left(\mathbf{X}^{\mathbb{K}}_{t+1} = i_{t+1}\right) + (1-\mathbb{I}_{\mathcal{B}}(i_{t+1}))p^a_{i_{t+1}}\mathbb{P}_{i_t}\left(\mathbf{X}^{\mathbb{K}}_{t+1} = i_{t+1}\right)\right] && (72)\end{aligned}$$

where $\mathbb{I}_{\mathcal{B}}(k)$ equals 1 if $k \in \mathcal{B}$ and 0 otherwise.

If $i_t \in \mathcal{B}$, then $B_s \subset A$, for $0 \leq s \leq t$, and

$$\begin{aligned}\frac{\mathbb{P}\left(\{\mathbf{X}^{\mathbb{K}}_{t+1} = i_{t+1}\} \cap B_s \cap A\right)}{\mathbb{P}(A \cap B_s)} &= \frac{\mathbb{P}\left(\{\mathbf{X}^{\mathbb{K}}_{t+1} = i_{t+1}\} \cap B_s\right)}{\mathbb{P}(B_s)} && (73)\\ &= \mathbb{P}\left(\mathbf{X}^{\mathbb{K}}_{t+1} = i_{t+1}|B_s\right) && (74)\\ &= \mathbb{P}\left(\mathbf{X}^{\mathbb{K}}_{t+1} = i_{t+1}|\mathbf{X}^{\mathbb{K}}_t = i_t\right) && (75)\end{aligned}$$

Equations (72) and (75) imply that the ratio on the right hand side of equation (67) does not depend on s, for $0 \leq s \leq t$. In particular,

$$\begin{aligned}\mathbb{P}\left(\mathbf{X}^{\mathbb{C}}_{t+1} = i_{t+1}|\mathbf{X}^{\mathbb{C}}_t = i_t, \ldots, \mathbf{X}^{\mathbb{C}}_0 = i_0\right) &= \frac{\mathbb{P}\left(\{\mathbf{X}^{\mathbb{K}}_{t+1} = i_{t+1}\} \cap B_t \cap A\right)}{\mathbb{P}(A \cap B_t)} && (76)\\ &= \mathbb{P}\left(\mathbf{X}^{\mathbb{C}}_{t+1} = i_{t+1}|\mathbf{X}^{\mathbb{C}}_t = i_t\right). && (77)\end{aligned}$$

This prove that $\mathbf{X}^{\mathbb{C}}$ satisfies the Markov property.

The transition probabilities of the Markov chain $\mathbf{X}^{\mathbb{C}}$ follow from the equations above. For

$j \in \mathcal{T}$ and $i \notin \mathcal{B}$,

$$\mathbb{P}\left(\mathbf{X}_{t+1}^{\mathbb{C}} = j | \mathbf{X}_t^{\mathbb{C}} = i\right) = \frac{p_j^a p_{ji}^{\mathbb{K}}}{p_i^a}, \tag{78}$$

with the convention that $p_k^a = 1$ for $k \in \mathcal{B}$. And we have for $i, j \in \mathcal{B}$,

$$\mathbb{P}\left(\mathbf{X}_{t+1}^{\mathbb{C}} = j | \mathbf{X}_t^{\mathbb{C}} = i\right) = 1. \tag{79}$$

It follows form equations (78) and (79) that the transition probabilities of the Markov chain $\mathbf{X}^{\mathbb{C}}$ are given by the matrix $\mathbf{P}_{\mathbb{C}}$ defined in Section 3.2, i.e.

$$\mathbf{P}_{\mathbb{C}} = \left(\begin{array}{c|c} \mathbf{U}_{\mathbb{C}} & \mathbf{0} \\ \hline \mathbf{M}_{\mathbb{C}} & \mathbf{I}_{\beta} \end{array}\right) \tag{80}$$

where

$$\mathbf{U}_{\mathbb{C}} = \mathbf{D}_a \mathbf{U}_{\mathbb{K}} \mathbf{D}_a^{-1} \quad \text{and} \quad \mathbf{M}_{\mathbb{C}} = \mathbf{K}\mathbf{D}_a^{-1}, \tag{81}$$

where $\mathbf{D}_a = \text{diag}\left(\mathbf{p}_a\right)$ is a diagonal matrix with, on the diagonal, the probabilities of absorption in the target states.

A.3 Moments of occupancy times

To calculate the moments of the occupancy time for individuals initially outside the target set $\mathcal{B}$, we use the strong Markov property. If the individual never enters in $\mathcal{B}$, then its occupancy time is zero. If it does enter in $\mathcal{B}$, say through the state j, then the law of its occupancy time is equal to the law of the occupancy time for an individual starting in the state j. To fix the

idea, consider the state $i \in \mathcal{B}^c$. Then

$$\begin{aligned}
\mathbb{E}(\tau_i^m) &= \sum_{\ell=1}^{\infty} \ell^m \mathbb{P}(\tau_i = \ell) \\
&= \sum_{\ell=1}^{\infty} \ell^m \left(\sum_{j\in\mathcal{B}} \mathbb{P}(\tau_i = \ell | \text{enter in } \mathcal{B} \text{ in } j) \mathbb{P}(\text{enter in } \mathcal{B} \text{ in } j) \right) \\
&= \sum_{\ell=1}^{\infty} \ell^m \left(\sum_{j\in\mathcal{B}} \mathbb{P}(\tau_j = \ell) a_{ji} \right) \\
&= \sum_{j\in\mathcal{B}} a_{ji} \sum_{\ell=1}^{\infty} \ell^m \mathbb{P}(\tau_j = \ell) \\
&= \sum_{j\in\mathcal{B}} a_{ji} \mathbb{E}(\tau_j^m), \qquad (82)
\end{aligned}$$

where a_{ji} is the probability that the killed Markov chain, starting in state i, is absorbed by the state j, as in Section 3.1.1. In matrix notation, equation (82) is equivalent to

$$\boldsymbol{\tau}_{\text{out}}^k = \mathbf{A}^\intercal \boldsymbol{\tau}_{\text{in}}^k. \qquad (83)$$

A.4 Covariance between the occupancy times in two disjoint sets

Here, we calculate the covariance between the occupancy time in two disjoint subsets $\mathcal{B}_1$ and $\mathcal{B}_2$, of the transient set $\mathcal{T}$. As stated in the main text, the covariance between $\boldsymbol{\tau}_{\mathcal{B}_1}$ and $\boldsymbol{\tau}_{\mathcal{B}_2}$ is

$$\text{Cov}\left(\boldsymbol{\tau}_{\mathcal{B}_1}, \boldsymbol{\tau}_{\mathcal{B}_2}\right) = \mathbb{E}\left[\left(\boldsymbol{\tau}_{\mathcal{B}_1} - \boldsymbol{\tau}_{\mathcal{B}_1}^1\right)\left(\boldsymbol{\tau}_{\mathcal{B}_2} - \boldsymbol{\tau}_{\mathcal{B}_2}^1\right)\right]. \qquad (84)$$

We rewrite the covariance between $\boldsymbol{\tau}_{\mathcal{B}_1}$ and $\boldsymbol{\tau}_{\mathcal{B}_2}$ in terms of their variances and the variance of their sum,

$$\text{Cov}\left(\boldsymbol{\tau}_{\mathcal{B}_1}, \boldsymbol{\tau}_{\mathcal{B}_2}\right) = \frac{1}{2}\left[\text{Var}\left(\boldsymbol{\tau}_{\mathcal{B}_1} + \boldsymbol{\tau}_{\mathcal{B}_2}\right) - \text{Var}\left(\boldsymbol{\tau}_{\mathcal{B}_1}\right) - \text{Var}\left(\boldsymbol{\tau}_{\mathcal{B}_2}\right)\right]. \qquad (85)$$

Since the sets $\mathcal{B}_1$ and $\mathcal{B}_2$ are disjoint, the occupancy time in the union $\mathcal{B}_1 \cup \mathcal{B}_2$ is the sum of the occupancy times in each of the subsets. Thus,

$$\text{Cov}\left(\boldsymbol{\tau}_{\mathcal{B}_1}, \boldsymbol{\tau}_{\mathcal{B}_2}\right) = \frac{1}{2}\left[\text{Var}\left(\boldsymbol{\tau}_{\mathcal{B}_1\cup\mathcal{B}_2}\right) - \text{Var}\left(\boldsymbol{\tau}_{\mathcal{B}_1}\right) - \text{Var}\left(\boldsymbol{\tau}_{\mathcal{B}_2}\right)\right]. \qquad (86)$$

The variances $\text{Var}\left(\boldsymbol{\tau}_{\mathcal{B}_1\cup\mathcal{B}_2}\right)$, $\text{Var}\left(\boldsymbol{\tau}_{\mathcal{B}_1}\right)$, and $\text{Var}\left(\boldsymbol{\tau}_{\mathcal{B}_2}\right)$ are calculated with the formulae (24) and (26) applied to the sets $\mathcal{B}_1 \cup \mathcal{B}_2$, $\mathcal{B}_1$, and $\mathcal{B}_2$, respectively.

A.5 One-step transition probabilities from $\mathcal{B}$ given return

Let w_{ij}^{in} be the conditional probability that an individual in target state $\alpha + j$ moves to the target state $\alpha + i$, in one time-step, given that it eventually returns to the target set. Then,

$$\begin{aligned} w_{ji}^{\text{in}} := \mathbb{P}_{\alpha+i}\left(X_1 = \alpha + j | T < \infty\right) &= \frac{\mathbb{P}_{\alpha+i}\left(X_1 = \alpha + j, T < \infty\right)}{\mathbb{P}_{\alpha+i}\left(T < \infty\right)} \\ &= \frac{\mathbb{P}_{\alpha+i}\left(X_1 = \alpha + j\right)}{\mathbb{P}_{\alpha+i}\left(T < \infty\right)} \\ &= \frac{u_{\alpha+j,\alpha+i}}{\sum_{\ell\in\mathcal{B}} u_{\ell i}^{\mathcal{B}}} \\ &= \frac{u_{\alpha+j,\alpha+i}}{p_i^r}, \end{aligned} \tag{87}$$

where $\mathbf{p}_r$ describes the return probabilities, as defined in equation (47). Thus,

$$\mathbf{W}_{\text{in}} = \mathbf{Q}\mathbf{D}_r^{-1} \quad \text{of size } \beta \times \beta, \tag{88}$$

where $\mathbf{D}_r = \text{diag}\left(\mathbf{p}_r\right)$ and the matrix $\mathbf{Q}$ is extracted from the matrix $\mathbf{U}$, as in equation (7). Let w_{ij}^{out} be the conditional probability that an individual in target state $\alpha + j$ moves to the non-target state i, in one time-step, given that it eventually returns to the target set. Then

$$\begin{aligned} w_{ji}^{\text{out}} := \mathbb{P}_{\alpha+i}\left(X_1 = j | T < \infty\right) &= \frac{\mathbb{P}_{\alpha+i}\left(T < \infty | X_1 = j\right)\mathbb{P}_{\alpha+i}(X_1 = j)}{\mathbb{P}_{\alpha+i}\left(T < \infty\right)} \\ &= \frac{\mathbb{P}_{\alpha+i}(X_1 = j)\mathbb{P}_{\alpha+j}\left(T < \infty\right)}{\mathbb{P}_{\alpha+i}\left(T < \infty\right)} \\ &= \frac{u_{j,\alpha+i}\sum_{\ell\in\mathcal{B}} a_{\ell j}}{\sum_{\ell\in\mathcal{B}} u_{\ell i}^{\mathcal{B}}} \\ &= \frac{u_{j,\alpha+i}p_j^a}{p_i^r}, \end{aligned} \tag{89}$$

where the vector p_a describes the probabilities of absorption in the target states, as defined in eqn. (11). Thus,

$$\mathbf{W}_{\text{out}} = \mathbf{D}_a\mathbf{L}\mathbf{D}_r^{-1} \quad \text{of size } \alpha \times \beta, \tag{90}$$

where $\mathbf{D}_r = \text{diag}\left(\mathbf{p}_r\right)$, $\mathbf{D}_a = \text{diag}\left(\mathbf{p}_a\right)$, and the matrix $\mathbf{L}$ is extracted form the matrix $\mathbf{U}$, as in equation (7).

Now, we derive the moments of $\boldsymbol{\mu}$, conditional on the individual returning to the target set.

Let $\alpha + i$ be a target state. Then

$$\begin{aligned}
\mathbb{E}[\mu_i^k | T < \infty] &= \sum_{n=1}^{\infty} n^k \mathbb{P}(\mu_i = n | T < \infty) && (91)\\
&= \mathbb{P}(\mu_i = 1 | T < \infty) + \sum_{n=2}^{\infty} n^k \sum_{j \in \mathcal{B}^c} \mathbb{P}(t_j^{\mathcal{B}} = n-1) \mathbb{P}_i(X_1 = j | T < \infty) && (92)\\
&= \mathbb{P}(\mu_i = 1 | T < \infty) + \sum_{j \in \mathcal{B}^c} \mathbb{P}_i(X_1 = j | T < \infty) \sum_{n=1}^{\infty} (n+1)^k \mathbb{P}(t_j^{\mathcal{B}} = n) && (93)\\
&= \mathbb{P}(\mu_i = 1 | T < \infty) + \sum_{j \in \mathcal{B}^c} \mathbb{P}_i(X_1 = j | T < \infty) \sum_{n=1}^{\infty} \sum_{r=0}^{k} \binom{k}{r} n^r \mathbb{P}(t_j^{\mathcal{B}} = n) && (94)\\
&= \mathbb{P}(\mu_i = 1 | T < \infty) + \sum_{j \in \mathcal{B}^c} \mathbb{P}_i(X_1 = j | T < \infty) \sum_{r=0}^{k} \binom{k}{r} t_{\mathcal{B}_j}^r && (95)\\
&= 1 + \sum_{j \in \mathcal{B}^c} w_{ji}^{\text{out}} \sum_{r=1}^{k} \binom{k}{r} t_{\mathcal{B}_j}^r. && (96)
\end{aligned}$$

Hence, in matrix notation,

$$\mathbb{E}[\boldsymbol{\mu}^k | T < \infty] = \mathbf{1}_\beta + \sum_{r=1}^{k} \binom{k}{r} \mathbf{W}_{\text{out}}^\intercal \mathbf{t}_{\mathcal{B}}^r. \qquad (97)$$

B Supplementary Material

1. Figues S1 and S2 are provided.

2. We provide the MATLAB files (see below) to carry out the calculations presented the Example.

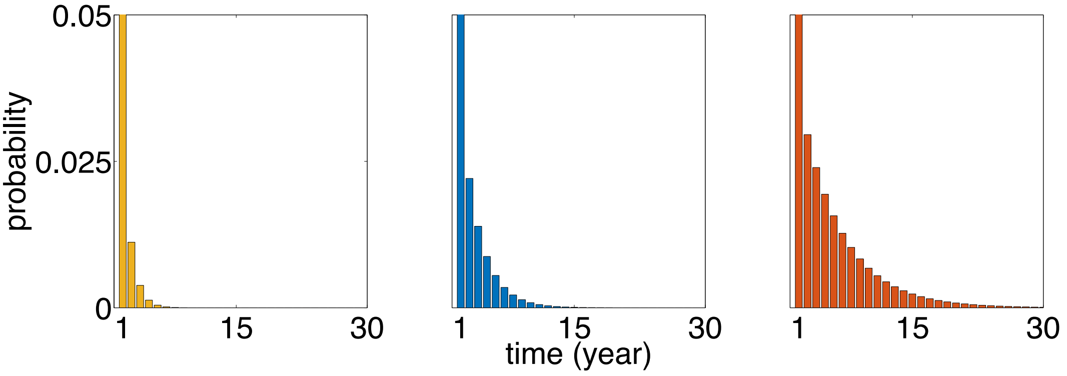

Figure S1: Distribution of the time required to return to the set $\mathcal{B}_\mathrm{b}$ for an individual initially in the state successful breeder, under favourable condition (left), ordinary condition (centre), and unfavourable condition (right). The y-axis is truncated at 0.05 to enhance the readability of the plots. The probability that the return time equals 1 is 0.983 under favorable ice conditions, 0.9403 under ordinary ice conditions, and 0.8444 under unfavorable ice conditions.

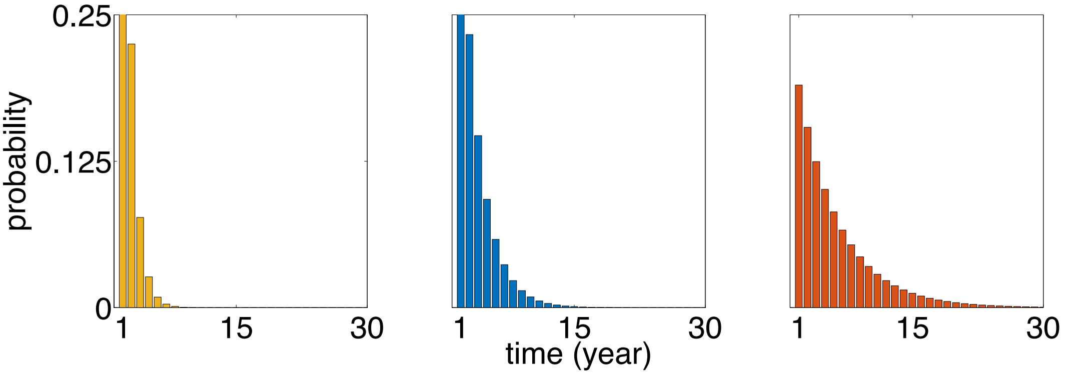

Figure S2: Distribution of the time required to reach the set $\mathcal{B}_\mathrm{b}$ for an individual initially in the state non breeder, under favourable condition (left), ordinary condition (centre), and unfavourable condition (right). The y-axis is truncated at 0.25 to enhance the readability of the plots. The probability that the reaching time equals 1 is 0.658 under favorable ice conditions, 0.37 under ordinary ice conditions, and 0.19 under unfavorable ice conditions.
