# Supplementary material for: Occupancy time in sets of states for demographic models
Source: Theor Popul Biol. 2018 Mar;120:62–77. doi: 10.1016/j.tpb.2017.12.007 (PMC5861321; doi:10.1016/j.tpb.2017.12.007)
Supplement: MMC S2 — This file contains the MATLAB codes to carry out the calculations presented in this paper for any transition matrix and any target set. This file contains also the transition matrices used in the Example. [file mmc2.pdf]

```
%created for use in occupancy time's paper Section Example: Southern
%Fulmar

%This script provides the transition matrices U_fav, U_ord, and U_unfav for
%favourable, ordinary, and unfavourable ice-conditions, respectively.

%For the original data, see
%S. JENOUVRIER et al (2015), Ecological Monographs 85:605?624.


%10/04/2017

U_fav=[0.828,0,0,0
0.06624,0.72912,0.62244,0.40176
0.02576,0.18228,0.24206,0.15624
0,0.0186,0.0455,0.342];


U_ord=[0.9016,0,0,0
0.011408,0.66737,0.49312,0.1809
0.006992,0.18823,0.24288,0.0891
0,0.0744,0.184,0.63];


U_unfav=[0.9154,0,0,0
0.002392,0.4873,0.25147,0.0468
0.002208,0.1895,0.23213,0.0432
0,0.2632,0.4464,0.81];
```

Published with MATLAB® R2013b

```
%Created for use in the paper "Occupancy time in sets of states
%for demographic models"

%Gregory Roth and Hal Caswell
%10/04/2017

%this function calculates the results of a set analysis in a Markov chain
%demographic model.

%Given:
%
%    - U                          a transient matrix
%    - B                          a target set
%
%Returns:
%
    %Occupancy time in the target set B

%    - out.tau                  mean
%    - out.tau2                 second moment
%    - out.vartau               variance
%    - out.cv_tau               coefficient of variation

    %Reaching the target set B

%    - out.p_a                    probability to reach
%    - out.t_B                    mean time to reach
%    - out.vart_B                 variance of time ot reach

    %Returning to the target set B

%    - out.p_r                    probability to return
%    - out.lambda                 mean time to return
%    - out.varlambda              variance time ot return

    %Useful matrices

%    - out.U_B                    transient matrix of the sub MC
%    - out.U_c                    transient matrix of the conditional MC
%    - out.U_k                    transient matrix of the killed MC


%************************************************************************

function out=occ_set(U,B)
siz=size(U);
s=siz(1);                    %size of U

sa=length(B);                %number of states in the target set B
st=s-sa;                     %number of states in the complement of B
```

```
%useful vectors
es=ones(1,s);
esa=ones(1,sa);
est=ones(1,st);

%permutation vector, used to rearrange the matrix U
p=(1:s);
for i=1:sa
    p(p==B(i))=[];
end
p=[p,B];

%Rearranging the indices of the transient states such that the last
%states are the states in B
        Utemp=U;
        for i=1:s
            Utemp(:,i)=U(:,p(i));
        end
        Utemp2=Utemp;
        for i=1:s
        Utemp2(i,:)=Utemp(p(i),:);
        end
        Uprime=Utemp2; %rearranged transition matrix



%Decomposing the transition matrix and computing useful matrices

        U_k=Uprime(1:st,1:st);  %transitions from B^c to B^c
        K=Uprime(st+1:end,1:st);%transitions from B^c to B
        L=Uprime(1:st,st+1:end);%transitions from B to B^c
        Q=Uprime(st+1:end,st+1:end);%transitions from B to B

        U_sub=Uprime(:,st+1:end); %transition probabilities from states in
                                  %B to any states (except death)

        N=inv(speye(s)-U); %fundamental matrix of the original
                           %chain (non rearranged)


%creating the killed Markov chain
        %transient matrix
          %U_k=U_k

         %fundamental matrix
         N_k=inv(eye(st)-U_k);

%Absorbtion probabilities, via the killed MC

        A_k=K*N_k;         %apbsorbtion probabilities
        p_a=esa*A_k;       %probabilities of abosrbition in B

%creating the conditonal Markov chain
         D_a=diag(p_a);
```

```
        %transient matrix and mortality matrix
         U_c=(D_a*U_k)/D_a;

         %fundamental matrix
         N_c=inv(speye(st)-U_c);

%creating the sub Markov chain

        %transient matrix
        U_B=Atilde_k*U_sub;

        %fundamental matrix
        N_B=inv(speye(sa)-U_B);

%computing the measures

        %Occupancy time in B

        tau_B=sum(N_B);
        tau2_B=esa*N_B*(2*N_B-eye(sa));

        tau=tau_B*Atilde_k;
        tau2=tau2_B*Atilde_k;
        vartau=tau2-tau.*tau;

        %Reaching the subset B

        t_B=est*N_c;          %mean time to reach
        t2_B=est*N_c*(2*N_c-eye(st));
        vart_B=t2_B-t_B.*t_B;

        %Returning to the subset B

        p_r=esa*U_B;  %return probabilities
        D_r=diag(p_r);

        W_out=(D_a*L)/D_r;   %conditional transition probabilities B to
                             %B^c given individual returns in B
        W_in=(Q)/D_r;        %conditional transition probabilities B to
                             %B given individual returns in B
        %return time
        lambda=esa+t_B*W_out;    %mean
        varlambda=t2_B*W_out-(t_B*W_out).*(t_B*W_out); %variance


%rearrange the output vectors in the initial order

        q=(1:s);
        for i=1:s
            q(i)=find(p==i);
        end

        tautemp=tau;
```

```
        tau2temp=tau2;
        vartautemp=vartau;

        for i=1:s
            tautemp(i)=tau(q(i));
            tau2temp(i)=tau2(q(i));
            vartautemp(i)=vartau(q(i));
        end

%outputs


out.tau=tautemp;
out.tau2=tau2temp;
out.vartau=vartautemp;
out.cv_tau= sqrt(vartautemp)./tautemp;

out.p_a=p_a;
out.t_B=t_B;
out.vart_B=vart_B;

out.p_r=p_r;
out.lambda=lambda;
out.varlambda=varlambda;

out.U_B=U_B;
out.U_c=U_c;
out.U_k=U_k;
end
```

Published with MATLAB® R2013b

```
%created for use in occupancy time's paper Section Example: Southern
%Fulmar

%Gregory Roth and Hal Caswell
%10/04/2017


%This code calculates the covariance and correlation between the occupancy
%time in the target set {successful breeder, failed breeder} and the
%longevity

%ATTENTION: this code uses the function abs_MC

%upload the trensient matrix U



T=[1,2,3,4]; %transient set
B=[2,3]; %target set={successful beeder, failed breeder}
Bc=[1,4]; %complement of the target set

temp1=abs_MC(U,T);
temp2=abs_MC(U,Bc);
temp3=abs_MC(U,B);

%covariance between occupancy in B and Bc
tempcov=.5*(temp1.vartau-temp2.vartau-temp3.vartau);

%covariance between occupancy in B and longevity
cov=tempcov+temp3.vartau;

%correlation between occupancy in B and longevity (eq. 38)
cor=cov./(sqrt(temp1.vartau.*temp3.vartau));
```

Published with MATLAB® R2013b
